# Terpyridine-functionalized single-walled carbon nanotubes towards selectivity in the oxygen reduction reaction†

**DOI:** 10.1039/d5na00281h

**Published:** 2025-06-04

**Authors:** Ioanna K. Sideri, Raul Arenal, Nikos Tagmatarchis

**Affiliations:** a National Hellenic Research Foundation, Theoretical and Physical Chemistry Institute 48 Vassileos Constantinou Avenue 11635 Athens Greece isideri@eie.gr tagmatar@eie.gr; b Instituto de Nanociencia y Materiales de Aragon (INMA), CSIC-U. de Zaragoza 50009 Zaragoza Spain; c Laboratorio de Microscopias Avanzadas (LMA), Universidad de Zaragoza 50018 Zaragoza Spain; d ARAID Foundation 50018 Zaragoza Spain

## Abstract

Single-walled carbon nanotubes (SWCNTs) have been widely employed as electrocatalysts due to their exceptional mechanical strength and electronic properties, despite lacking intrinsic catalytic centers. Current research efforts focus on enhancing their catalytic activity by modifying them with organometallic chelate complexes, to achieve controlled molecular-level structuring and improved performance in Proton Exchange Membrane Fuel Cells (PEMFCs). While the mechanistic understanding of their electrocatalytic behavior in relation to molecular structure remains in its early stages, it offers valuable insights for the development of efficient electrocatalysts. In this study, SWCNTs were stepwise chemically modified with terpyridine ligands both in the absence of metal and in the presence of ruthenium (Ru) in two different oxidation states. The resulting SWCNT-based nanomaterials were characterized using Raman, UV-Vis, XPS and energy-dispersive X-ray spectroscopy (EDS) coupled with transmission electron microscopy (TEM) imaging, while the degree of functionalization was assessed through thermogravimetric analysis (TGA). A comprehensive electrocatalytic investigation demonstrated how covalent modification with Ru^2+^ and Ru^3+^ terpyridine complexes influences the mechanistic pathway of the oxygen reduction reaction (ORR). This comparative analysis underscores the critical role of the first coordination sphere of precious metals in both the thermodynamics and kinetics of ORR, when anchored onto carbon nanomaterial lattices, providing valuable insights for the nanostructured design of efficient carbon-based electrocatalysts.

## Abstract 2

A detailed study on the effect of different oxidation states of ruthenium-metalated terpyridine-functionalized single walled carbon nanotubes (SWCNTs) on the oxygen reduction reaction (ORR) activity and selectivity.

## Graphical abstract

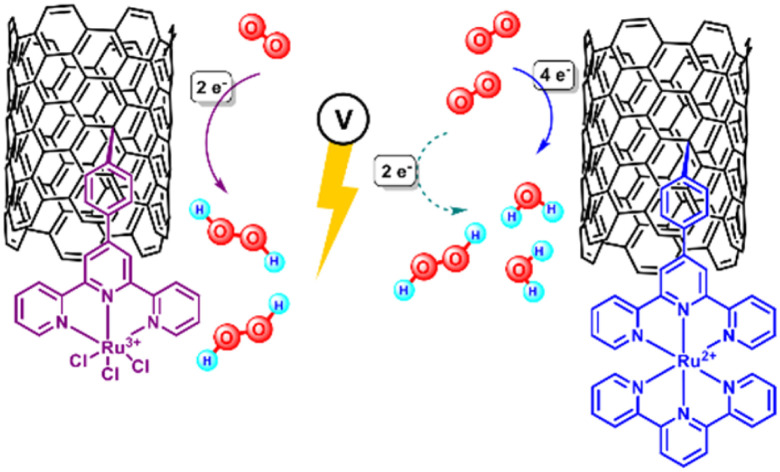

## 1. Introduction

The oxygen reduction reaction (ORR) constitutes a fundamental process for various clean energy technologies and industrial applications. However, certain limitations exist that hamper the ORR applicability in new technologies, which are the low selectivity and the sluggish reaction kinetics of the electrocatalysts developed. Understanding the mechanistic aspects of this complex electrocatalytic process is imperative, in order to design and develop efficient electrocatalysts. It is known that ORR proceeds *via* two competing mechanistic pathways: one involving a 2e^−^ transfer, producing H_2_O_2_ as the final reduction product, and the other involving a 4e^−^ transfer, leading to the production of H_2_O. Both mechanistic pathways are important for different application scope; while the 4e^−^ mechanism is generally preferred, as the formation of H_2_O is more efficient and environmentally friendly than H_2_O_2_ production, the 2e^−^ pathway is particularly useful in the hydrogen peroxide production industry. From a mechanistic point of view, the discussion is evolved around the catalytic ability of adsorption and desorption of *OH and *OOH intermediates, whether it is a four-electron associative/dissociative process or a two-electron dissociative pathway.^1–3^ The catalytic ability of the catalyst to adsorb *OOH is crucial in determining whether a 2e^−^ or 4e^−^ ORR occurs.^4,5^

ORR electrocatalysts range from the traditional platinum-based catalysts to later transition metal catalysts, followed by carbon-based catalysts,^6–8^ single-atom catalysts, *etc.*^9,10^ Carbon nanostructures have been extensively used as electrocatalysts for proton exchange membrane fuel cells (PEMFCs) due to their high mesoporosity, surface area, electrical conductivity and stability under harsh oxidizing conditions.^11,12^ Despite not possessing intrinsic catalytic activity, carbon nanostructures owe their applicability to their functionalization capability. In particular, single-walled carbon nanotubes (SWCNTs) have attracted significant attention as electrocatalyst supports for the oxygen reduction reaction (ORR) due to their high electrical conductivity, structural uniformity, and large surface area.^13–15^ Numerous studies have demonstrated that SWCNTs, particularly when modified or combined with metal or heteroatom functional groups, can promote favorable adsorption of O_2_ and enhance electron transfer kinetics.^13,16,17^ Their one-dimensional structure facilitates efficient charge transport, while their tunable electronic states – especially in semiconducting chiralities like (7,6) – allow fine control over the interaction with oxygen intermediates.^18^

On the other hand, molecular complexes owe their electrocatalytic activity to the link between their structure and function, are often bio-inspired catalysts and exhibit good activity while also providing mechanistic insights relevant to synthetic and enzymatic catalysts.^19^ However, the use of molecular electrocatalysts in heterogeneous catalytic systems and aqueous environments is rather challenging due to incompatibility, instability or limited solubility. In this context, the scientific community has aimed to develop modified carbon nanostructures decorated with molecular complexes in a way that maintains or even enhances the catalytic activity of these electrocatalysts, while at the same time broadens their application scope. The covalent anchoring of metal chelating compounds onto carbon nanostructures for the preparation of heterogeneous electrocatalysts is a bright example of this exact tactic. The covalent functionalization allows full control over the chemical structure of nano-electrocatalysts at the molecular level and enables the materials to acquire desirable electrocatalytic properties, facilitating the unobstructed transfer of electrons across their surface. Such chelating ligands include terpyridines, which have the ability -depending on their structure and chemical environment- to bind bidentately or tridentately to metal centers, allowing them to play a key role in numerous catalytic applications.^20^

In particular, terpyridine complexation with Ru has been extensively utilized for modifying carbon nanotubes and MoS_2_ to develop catalysts for the electrochemical water-splitting reaction, which involves hydrogen evolution reaction (HER) and oxygen evolution reaction (OER).^21–26^ However, an in-depth understanding of the action mechanism of the metal, depending on the electrocatalytic reaction (HER or OER) during water splitting, remains in its early stages, despite being crucial for designing efficient catalysts for each electrochemical reaction. Regarding OER, significant efforts have been made in this direction, suggesting that the reaction mechanism is initiated by the presence of Ru^2+^ and its oxidation to Ru^3+^.^24,26^ In contrast, similar studies have not yet been conducted for the oxygen reduction reaction (ORR). Therefore, further exploration of the mechanisms, thermodynamics and kinetics, involved in the ORR process in terms of molecular engineering in heterogeneous electrocatalysis, investigating the differentiation between the 2e^−^ ORR and 4e^−^ ORR in this process are needed, in order to guide the design and development of efficient catalysts with high-selectivity to either H_2_O_2_ or H_2_O.

Herein, the stepwise chemical modification of single-walled carbon nanotubes (SWCNTs) with terpyridines in the absence of metal, as well as in the presence of Ru in two different oxidative states takes place, while thorough investigation is conducted on whether and how the oxidation state determines the ORR mechanistic pathway. (7,6)-SWCNTs were chosen based on their ability to undergo covalent functionalization without significant disruption of their π-conjugated framework. This makes them particularly suitable for anchoring coordination complexes or catalytically active species while preserving electrical connectivity.^27,28^ Functionalized SWCNT-based systems have been shown to outperform many traditional carbon supports in terms of ORR activity and durability in alkaline media.^15,17,29^ Thus, our choice to use (7,6)-SWCNTs modified with terpyridine-Ru complexes, aims to combine tailored electronic properties with robust molecular functionalization strategies, addressing a gap in literature related to the rational design of SWCNT-based ORR electrocatalysts. Extensive electrocatalytic study reveals the impact of covalent modification on the lattice of SWCNTs with terpyridine complexes of Ru^2+^ and Ru^3+^ on the mechanistic pathway of ORR. This comparative study highlights the importance of the first sphere of substituents of precious metals on both the thermodynamics and kinetics of ORR when anchored on the lattice of carbon nanomaterials, therefore provides valuable insights towards the nanostructure design of carbon-based effective electrocatalysts.

## 2. Experimental

### 2.1 Materials

All solvents and reagents were commercially available and used without further purification unless stated otherwise.

#### 2.1.1 Preparation of functionalized (7,6)-SWCNT-based material 1

For the chemical modification of carbon nanotubes *via* diazonium salts, a modified literature-based approach was followed.^30^ In a two-neck round-bottom flask, pristine (7,6)-SWCNTs (5 mg) were transferred and dispersed in *ortho*-dichlorobenzene (6 mL). The mixture was sonicated for 10 minutes. Then, terpyridine (1.6 mmol, 540 mg), dissolved in acetonitrile (3 mL), was added. A vertical condenser and an N_2_-filled balloon were attached, and the system was degassed three times using a vacuum line. During the final N_2_ filling cycle, the balloon was partially filled to allow space for the expansion of N_2_ released during the reaction. Finally, isoamyl nitrite (2.4 mmol, 280 mg) was added using a syringe, and the reaction was placed in an oil bath at 60 °C for 16 hours. The reaction mixture was then filtered under vacuum using a PTFE filter, followed by extensive washing with *N*,*N*-dimethylformamide and chloroform. Intermediate sonication treatments were performed to ensure the complete removal of any physiosorbed organic compounds until the filtrate was completely decolorized, as verified by absorption spectroscopy. The isolated black solid (5.5 mg) was dried under high vacuum.

#### 2.1.2 Preparation of modified (7,6)-SWCNT-based material 2

In a round-bottom flask containing material 1 (5 mg) and the RuCl_3_·H_2_O complex (1 mg), ethanol is added, and the reaction mixture is sonicated for 10 minutes before being stirred at room temperature for 2 hours. Subsequently, the reaction mixture is filtered under vacuum using a PTFE filter, and the solid residue is washed with ethanol and diethyl ether. Finally, material 2 is dried under high vacuum and isolated as a black powder (5.5 mg).

#### 2.1.3 Preparation of modified (7,6)-SWCNT-based material 3

Material 2 (5 mg) is transferred to a round-bottom flask, and terpyridine (2 mg) and ethylene glycol (1 mL) are added under a nitrogen (N_2_) atmosphere. The reaction mixture is sonicated and then heated in an oil bath at 130 °C for 16 hours under N_2_. Subsequently, the reaction mixture is filtered under vacuum using a PTFE filter, and the solid residue is thoroughly washed with ethanol and diethyl ether. Finally, material 3 is dried under high vacuum and isolated as a black powder (5 mg).

### 2.2 Catalyst preparation and deposition onto the working electrode

To prepare the catalyst ink, 4.0 mg of each material in powder form, were dispersed in a mixture of solvents (1 mL) containing water, isopropanol, and 5% Nafion (v/v/v = 4 : 1 : 0.02) and sonicated for 15 min. The working electrode was first cleaned through polishing using 6-, 3- and 1-mm diamond pastes, rinsed with deionized water, and sonicated in double-distilled water. Then, 8.5 μL aliquots of the catalyst ink were drop-casted on the electrode surface and dried at room temperature.

## Results and discussion

**Scheme 1:**
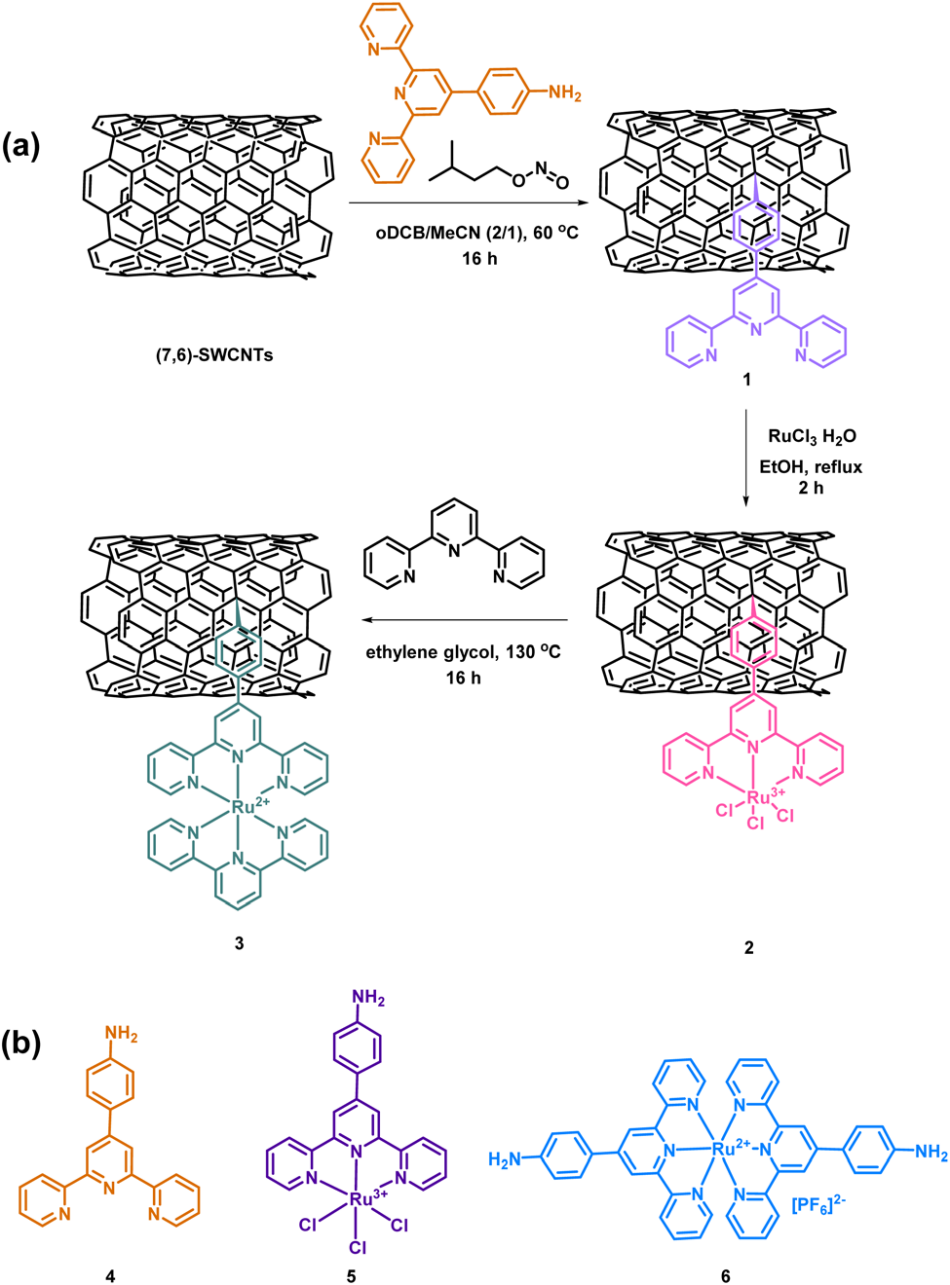
(a) Stepwise functionalization of (7,6)-SWCNTs with terpyridine substituents followed by Ru metalation. (b) Terpyridine substituents- organic analogues of the functionalized materials.

The terpyridine ligand utilized was synthesized according to the literature^31,32^ and was appropriately selected to bear an aniline precursor of an active aryl radical, which is added to the graphitic lattice of (7,6)-SWCNTs *via* a radical-nucleophilic aromatic substitution (Scheme 1).^30^

The attachment of the terpyridine ligand onto the SWCNT lattice led to the preparation of material 1 (Scheme 1). Subsequently, metalation of material 1 with RuCl_3_ was carried out, resulting in material 2, and the synthesis was completed by “capping” the metal complex with an additional terpyridine ligand, yielding material 3 (Scheme 1). The stepwise synthesis approach was chosen to allow complete spectroscopic and structural characterization of each intermediate, enabling comparative studies of intermediate and final materials concerning ORR. Specifically, during the transition from material 2 to 3, the Ru oxidation state decreases from +3 to +2, a parameter that could potentially affect the electrocatalytic reaction mechanism, assuming Ru serves as the active catalytic center. Additionally, organic analogs of materials 1, 2, and 3 were synthesized -compounds 4, 5, and 6, respectively (Scheme 1) (see ESI Section†)- to serve as reference molecules.

**Fig. 1:**
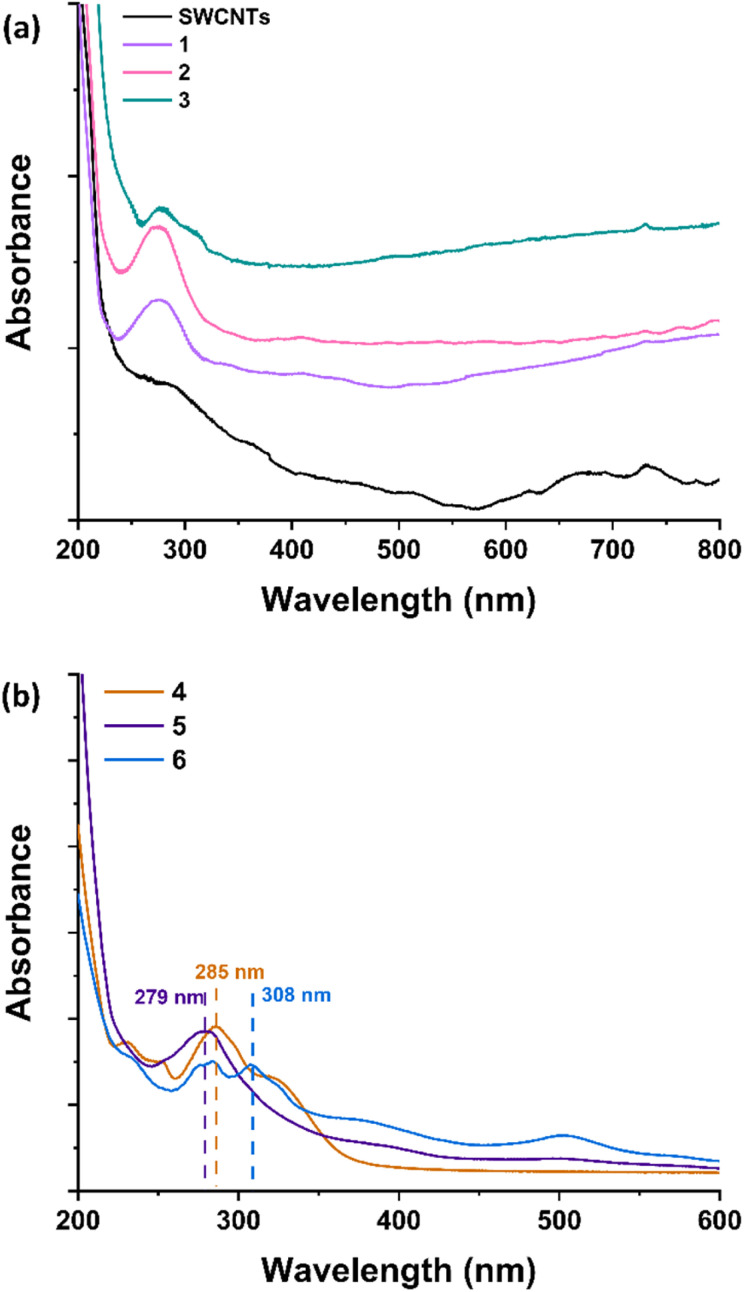
(a) UV-Vis spectra of (7,6)-SWCNTs (black) and functionalized materials 1 (purple), 2 (pink) and 3 (green). (b) Corresponding UV-Vis spectra of molecular analogues 4 (orange), 5 (deep purple) and 6 (light blue).

Confirmation of the successful chemical modification of SWCNTs with terpyridine ligands, as well as subsequent Ru metalation, was obtained *via* UV-Vis absorption spectroscopy. The UV-Vis spectra of pristine (7,6)-SWCNTs and materials 1, 2, and 3, and compounds 4, 5, and 6 are shown in Fig. 1. The terpyridine ligands 4 and 5 exhibit absorption maxima at 285 and 279 nm, respectively, while the Ru^2+^ complex 6 has an additional maximum at 308 nm (Fig. 1b). Observing the corresponding UV-Vis spectra of the materials, an absorption band at 278 nm for material 1 is identified and at 274 nm for material 2, while also an additional absorption band at 308 nm is observed, for the final material 3. The 7 nm hypsochromic shift between the absorption maximum of organic analogue 4 and material 1, and the respective 5 nm hypsochromic shift from absorption maximum of ligand 5 and material 2, implies the successful covalent modification. In parallel, the 4 nm absorption maximum shift between materials 1 and 2 confirms the successful metalation (Ru^3+^ complex formation) in accordance with the transition from ligand 4 to ligand 5. Finally, the additional absorption band at 308 nm in material's 3 UV-Vis profile, confirms the successful reduction to Ru^2+^ with the complexation of an additional terpyridine ligand (3), in agreement with the UV-Vis profile of organic complex 6 (Fig. 1a). Notably, SWCNTs possess intrinsic absorption bands attributed to van Hove singularities, which primarily appear at low energies and long wavelengths (>600 nm).^33–35^

**Fig. 2:**
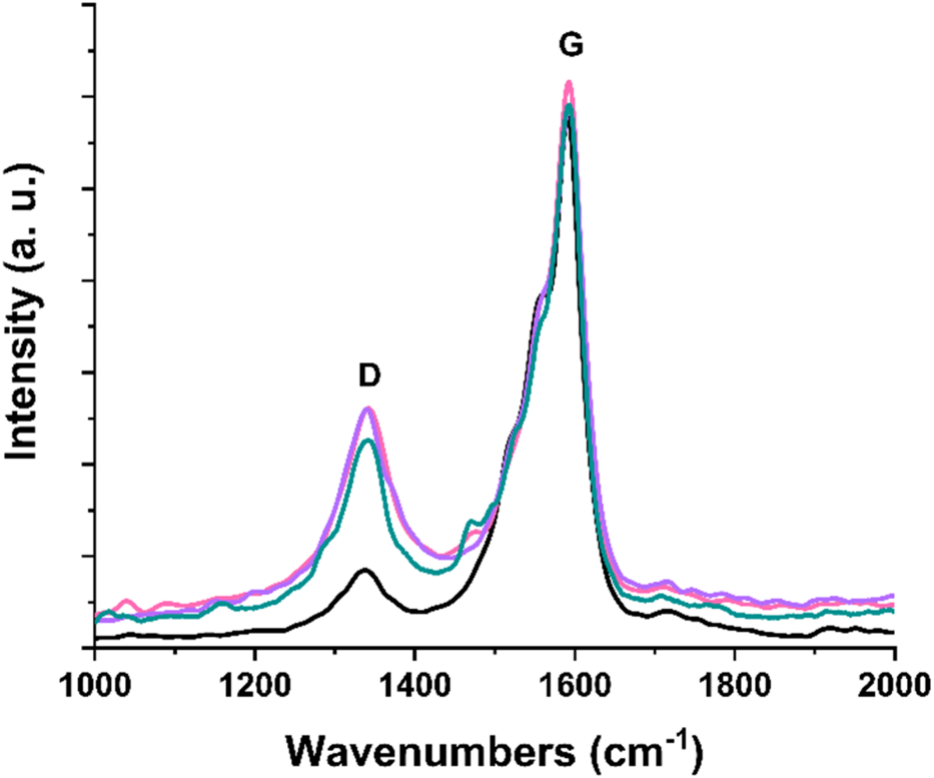
Raman spectra (514 nm) of (7,6)-SWCNTs (black), and functionalized materials 1 (purple), 2 (pink) and 3 (green).

Raman spectroscopy (514 nm), integral part of the spectroscopic characterization of carbon nanostructures, confirms the covalent modification that occurs through radical-nucleophilic aromatic substitution with the introduction of terpyridine substituents. The characteristic profile of carbon nanotubes, consisting of the D band (1340 cm^−1^) and G band (1590 cm^−1^), is evident in all Raman spectra of the (7,6)-SWCNTs and functionalized materials 1, 2, and 3 (Fig. 2). Following chemical functionalization of SWCNTs and subsequent normalization of all obtained spectra to the G band, the D band—associated with lattice defects—shows increased intensity due to the structural disturbance caused by the introduction of organic substituents.^36^

This is mirrored to a percentage rise in the hybridization of carbon atoms in the graphitic lattice of SWCNTs from sp^2^ (G band) to sp^3^ (D band) in the functionalized materials and is reflected in the increase of the *I*_D_/*I*_G_ intensity ratio upon chemical treatment. Specifically, this ratio rises from 0.18 for the commercially available (7,6)-SWCNTs to approximately 0.40 for the modified materials 1, 2, and 3.

**Fig. 3:**
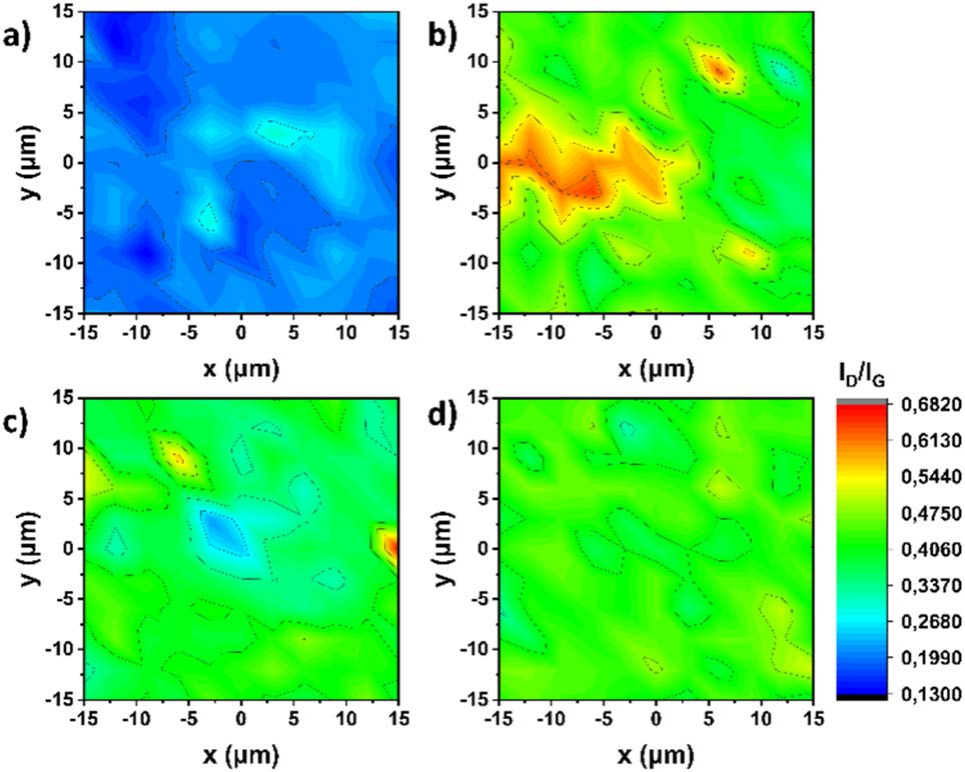
Raman spectroscopy (514 nm) mappings (scanning area: 30 μm × 30 μm) of (a) (7,6)-SWCNTs, and functionalized materials (b) 1, (c) 2, and (d) 3.

This change is visually represented in the color maps of Fig. 3, where the uniformity of the lattice is evident both before (Fig. 3a) and after the intervention on the sp^2^ lattice (Fig. 3b–d).

This observation constitutes direct evidence of the successful covalent modification realized on the surface of SWCNTs furnishing terpyridine-modified material 1. Regarding the subsequent stepwise engineering taking place for the preparation of metalated materials 2 and 3, no further interference on the sp^2^ lattice results in no change in the *I*_D_/*I*_G_ intensity ratio, as expected.

**Fig. 4:**
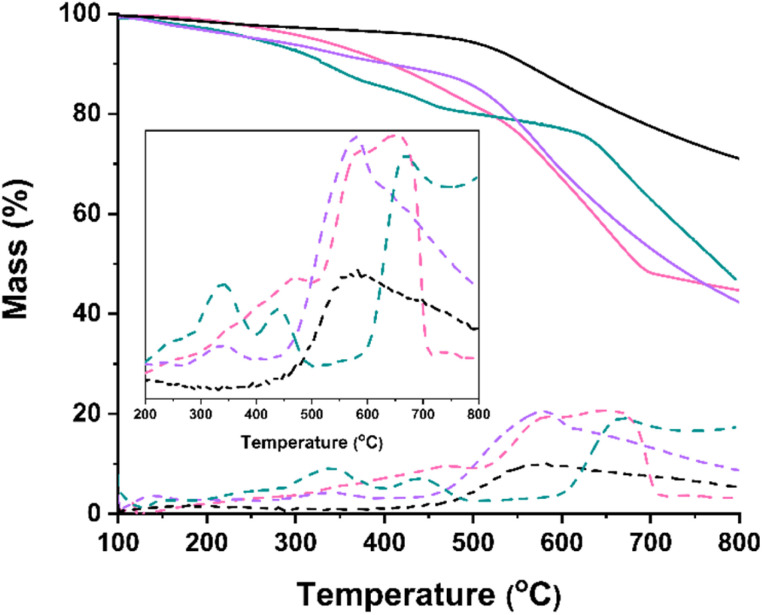
Thermogravimetric analysis (TGA) graphs recorded under N_2_ constant flow of (7,6)-SWCNTs (black), and functionalized materials 1 (purple), 2 (pink), and 3 (green). Dotted lines represent the 1st weight derivatives of each TGA curve. Inset displays a magnified area of the 1st weight derivatives of each material, clearly showcasing the degradation steps.

Having confirmed the covalent attachment of the substituents onto the carbon nanotube lattice and their uniform distribution, the determination of the degree of chemical modification achieved, is examined. Thermogravimetric analysis (TGA) (Fig. 4) serves as a valuable tool in this endeavor. It is observed that while the precursor (7,6)-SWCNT material remains thermally stable up to 500 °C under nitrogen (N_2_) atmosphere, the functionalized materials 1, 2, and 3 exhibit significant weight loss within the same temperature range, attributed to the thermal decomposition of the organic terpyridine chain. Specifically, a 9% weight loss is recorded for material 1, while materials 2 and 3 exhibit weight loss of 13% and 15%, respectively. In Fig. 4, the dotted lines represent the respective 1st weight derivatives for each material. It is evident that in the temperature range between 200–500 °C (Fig. 4, inset), while material 1 exhibits one degradation step at ∼340 °C, accounting for the decomposition of the terpyridine ligands, material 2 exhibits a poorly-defined degradation step shifted in higher temperature, revealing an increased degradation resistance, possibly owing to the Ru^3+^ complexation. Finally, material 3 exhibits two well defined degradation steps, one at 342 °C and a second one at 436 °C accounting for the Ru^2+^-terpyridine complex. Based on these findings and taking into account the molecular weights of the ligands in each case, it is roughly estimated that the SWCNT lattice is decorated with one organic substituent per 250 carbon atoms.

**Fig. 5:**
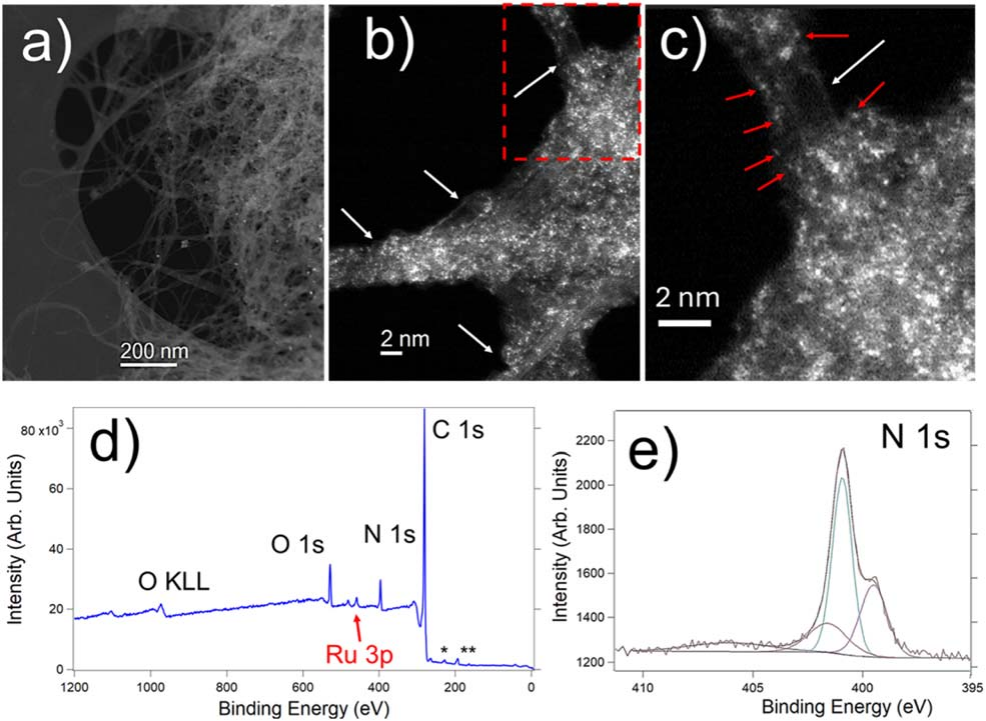
(a–c) HAADF-STEM images of terpyridine-functionalized SWCNTs-based material 3: (a) low-magnification, (b) and (c) high-magnification micrographs ((c) being a zoom of the red marked area of (b)), respectively. The SWCNTs (some of them highlighted in (b) and (c) by white arrows) and the Ru atoms (white dots in the images, some of them highlighted in (c) by red arrows) are visible in these micrographs. (d) XPS survey spectra of this sample, where C 1s, N 1s, O 1s and Ru 3p are observed. * and ** correspond to S and Cl impurities and they represent less than 1 at%. (e) High-resolution XPS N 1s spectrum, which has also been recorded in this system.

The combined use of macroscopic X-ray photoelectron spectroscopy (XPS) and local (sub-nanometer, even atomic scale) scanning transmission electron microscopy (STEM) provides a powerful approach for obtaining critical insights into both the structural and chemical composition of these complex materials.^37–39^Fig. 5 shows such kinds of investigations carried out on terpyridine-functionalized SWCNTs-based material 3. Fig. 5a corresponds to a low magnification image, where the existence of SWCNTs is observed. Additionally, Fig. 5b is a high-angle annular dark-field (HAADF) STEM image, where the SWCNTs are clearly depicted. A closer look reveals that the SWCNTs are covered by some material/sheath, which corresponds to Ru-terpyridine moieties. HAADF-STEM signal depends on the atomic number Z by approximately Z^1.7^.^40–42^ Thus, it is easy to attribute the atomic species observed in Fig. 5c image (white dots in the image, some of them highlighted by red arrows) to the ruthenium atoms of the terpyridines. This is also confirmed by HR-TEM imaging and energy-dispersive X-ray spectroscopy (EDS)-TEM, performed in these materials (Fig. S1†).

Further XPS analysis displayed in Fig. 5d and e confirms these findings. Fig. 5d shows a survey XPS spectrum recorded on the terpyridine-functionalized SWCNTs-based material 3. The existence of C, N, O and Ru is confirmed. High-resolution spectra of these elements have been acquired and this one of the N 1s is depicted in Fig. 5d. This N 1s spectrum can clearly be assigned to this one of terpyridine.^43^ Indeed, the peaks at 399.4 eV and 400.9 eV can be attributed to the contributions of the pyridinic nitrogen configuration and its bonding with Ru atoms, respectively.^44^ The elemental composition derived from this XPS spectrum is 84.4% C, 6.4% N, 5.9% O, and 1.3% Ru (atomic%). These results align well with the expected values for such system, confirming its composition. All these findings provide valuable insights into the configuration of these heterostructures, enabling a deeper understanding and further investigation of their structural and chemical properties.

**Fig. 6:**
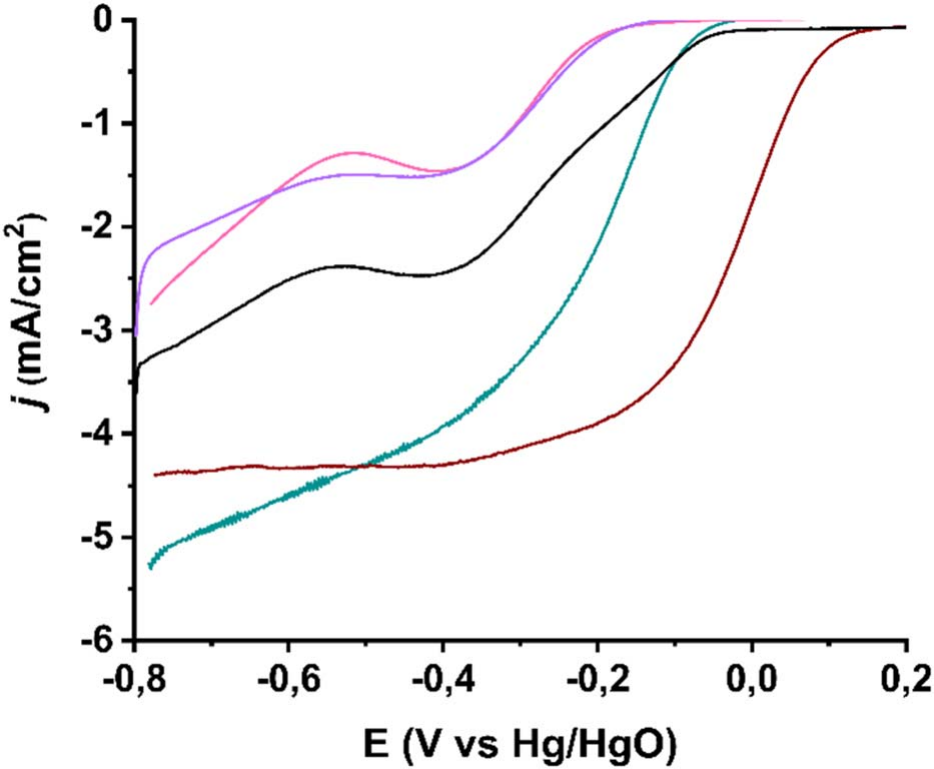
LSV ORR curves recorded with a rotation speed of 1600 rpm for (7,6)-SWCNTs (black), and functionalized materials 1 (purple), 2 (pink) and 3 (green), in comparison with the commercially available benchmark catalyst Pd/C (wine). All assays were performed in O_2_-saturated aqueous 0.1 M KOH as electrolyte with a scan rate of 5 mV s^−1^. The current densities (*j*) are normalized to the electrode surface.

To study the electrocatalytic activity for the oxygen reduction reaction (ORR), linear sweep voltammetry (LSV) measurements were performed using a rotating ring-disk electrode (RRDE) on (7,6)-SWCNTs, 1, 2, and 3 in an O_2_-saturated 0.1 M KOH electrolyte, at a rotation speed of 1600 rpm. Simultaneously, the ORR activity of the reference electrocatalyst Pd/C was recorded. Fig. 6 depicts the LSV curves recorded for (7,6)-SWCNTs and modified materials 1, 2, and 3. Initially, the precursor SWCNT material exhibits relatively good activity in terms of the onset potential (−56 mV *vs.* Hg/HgO). However, after chemical modification, this catalytic activity decreases. Specifically, materials 1 and 2 exhibit onset potentials of −144 mV and −164 mV, respectively, which are approximately 100 mV less positive than that of SWCNTs. Based on this first observation, it is implied that covalent functionalization of the SWCNT lattice negatively impacts ORR catalytic activity. This impediment arises as a consequence of the extended sp^2^ network disturbance with the interference of the sp^3^ carbon atoms after the nucleophilic substitution, which negatively impacts the voltage-induced conductivity of the material. It is noteworthy that the impact of the covalent modification of SWCNTs on the ORR activity is far from negligible despite the relatively scarce-dense terpyridine decoration achieved as evidenced by TGA. Subsequent metalation of the terpyridine substituent of 1 with Ru furnishing a Ru^3+^ complex (material 2) does not reverse this effect, despite Ru being well known for its excellent catalytic activity toward ORR.

However, by “capping” the metal complex with an additional terpyridine substituent, material 3 is obtained, which exhibits an improved onset potential of −36 mV *vs.* Hg/HgO-on average, ∼120 mV more positive than that of materials 1 and 2. Additionally, the half-wave potentials reflect the same trend. The final material 3 has a half-wave potential of −167 mV, while the two preceding steps exhibit values of −276 mV and −280 mV *vs.* Hg/HgO, respectively. The corresponding value for SWCNTs is −183 mV. This indicates an overall improvement of about 20 mV for material 3 in terms of both the half-wave potential and the onset potential, given the dramatic decrease in activity at the intermediate steps of the synthetic process. In Table S1,† the various electrocatalytic parameters of ORR deriving from the LSV curves, for all studied materials, are presented for comparison purposes.

Additionally, the diffusion-limited current density (*j*_d_) for material 3 reached 4.30 mA cm^−2^, while the kinetic current density (*j*_k_) was calculated only 0.22 mA cm^−2^ at −80 mV (*vs.* Hg/HgO) (Table S1†). Regarding *j*_d_, the value is significantly improved in the final material 3 compared to the precursor materials, which exhibit values ranging between 1.3–2.4 mA cm^−2^ (Table S1†). However, the kinetic currents were found to be very low for all the materials studied. Overall, the catalytic activity of the materials, from the most efficient to the least desirable, follows the order 3 > SWCNTs > 1 > 2. Table S1† summarizes the electrochemical behavior parameters of the studied electrocatalysts in comparison with commercially available Pd/C towards ORR. Notably, in the aforementioned ranking, the two metal-containing materials, 3 and 2, occupy the first and last positions, respectively. This observation highlights the importance of both the chemical structure of the first coordination sphere of the active catalytic center and the oxidation state of the metal substituent in the nanomaterial, as both factors strongly influence the reaction mechanism, which ultimately determines the overall catalytic activity.

**Fig. 7:**
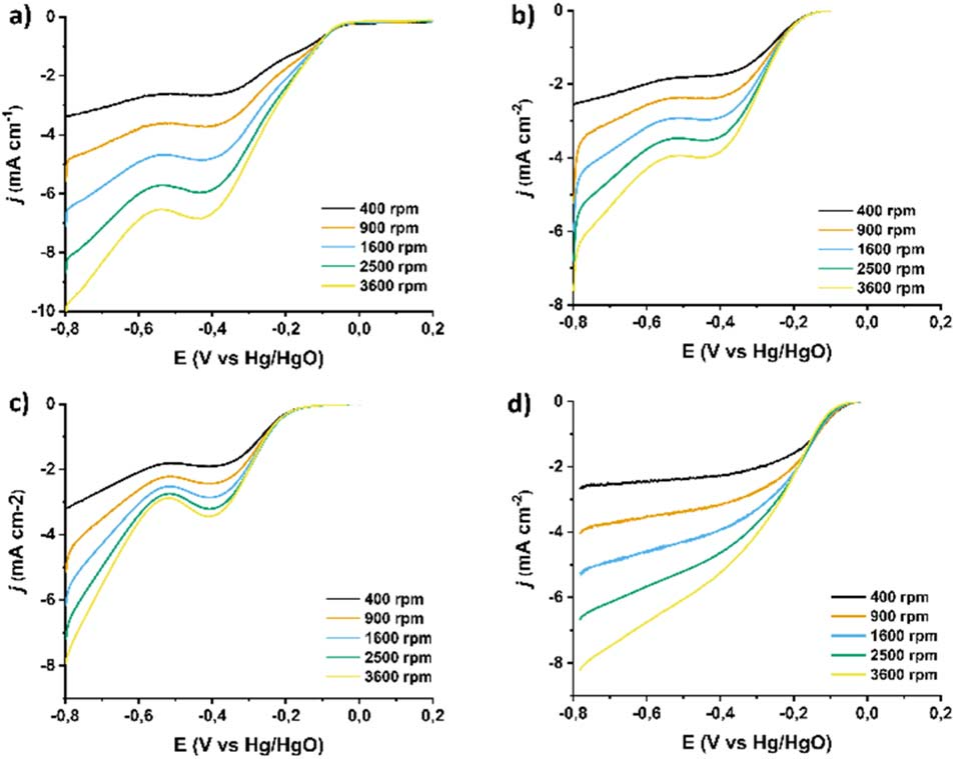
LSV ORR curves recorded with different rotation speeds (400–3600 rpm) for (a) (7,6)-SWCNTs, and functionalized materials (b) 1, (c) 2, and (d) 3. All assays were performed in O_2_-saturated aqueous 0.1 M KOH as electrolyte with a scan rate of 5 mV s^−1^. The current densities (*j*) are normalized to the electrode surface.

In order to evaluate this observation one step further, mechanistic studies were conducted in order to explore whether and how each functionalization step impacts the ORR mechanistic pathway (2e^−^*vs.* 4e^−^ mechanism) The reaction mechanism is inherently linked to its kinetics. To study the ORR kinetics, rotating ring disk electrode (RRDE) experiments were conducted by varying the rotation speed of the electrode. The corresponding LSV curves for ORR in pristine (7,6)-SWCNTs, 1, 2, and 3, recorded at rotation speeds ranging from 400 to 3600 rpm, are shown in Fig. 7a–d, respectively. These curves indicate a typical increase in current with increasing rotation speed due to the reduction of the diffusion layer at higher speeds.

**Fig. 8:**
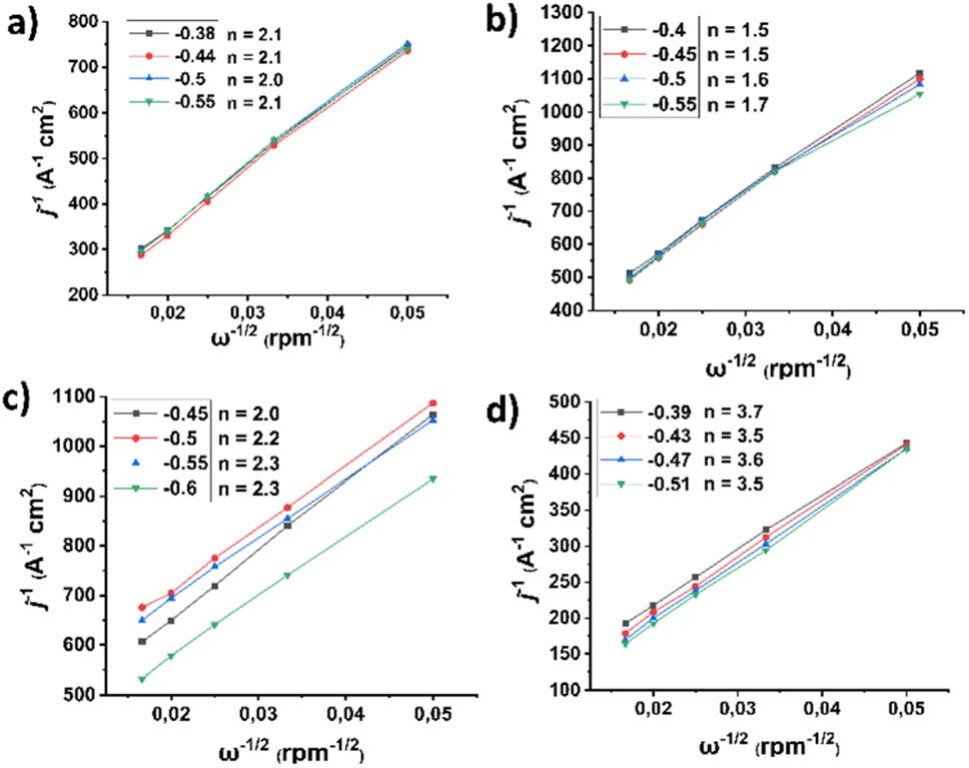
K–L plots of the ORR LSV curves recorded with different rotation speeds (400–3600 rpm) for (a) (7,6)-SWCNTs, and functionalized materials (b) 1, (c) 2, and (d) 3. All assays were performed in O_2_-saturated aqueous 0.1 M KOH as electrolyte with a scan rate of 5 mV s^−1^. The current densities (*j*) are normalized to the electrode surface.

Subsequently, the corresponding Koutecký–Levich (K–L) plots (Fig. 8) were constructed at different potentials based on the inverse of the current density (*j*^−1^) *versus* the inverse square root of the rotation speed (*ω*^−1/2^). As evident, these plots exhibit good linearity, indicating first-order kinetics with respect to the concentration of dissolved O_2_. Based on this analysis, the number of electrons transferred per oxygen molecule (*n*_KL_) was calculated to be 2.0–2.1, 1.5–1.7, 2.0–2.3, and 3.5–3.7 for pristine (7,6)-SWCNTs and functionalized materials 1, 2, and 3, respectively. Such an *n* value suggests a 2e^−^ mechanism for the precursor materials but a nearly 4e^−^ selectivity for the final material 3, indicating that for the latter the dominant reaction pathway involves direct reduction of dissolved O_2_ to H_2_O, bypassing the intermediate formation of H_2_O_2_.

**Fig. 9:**
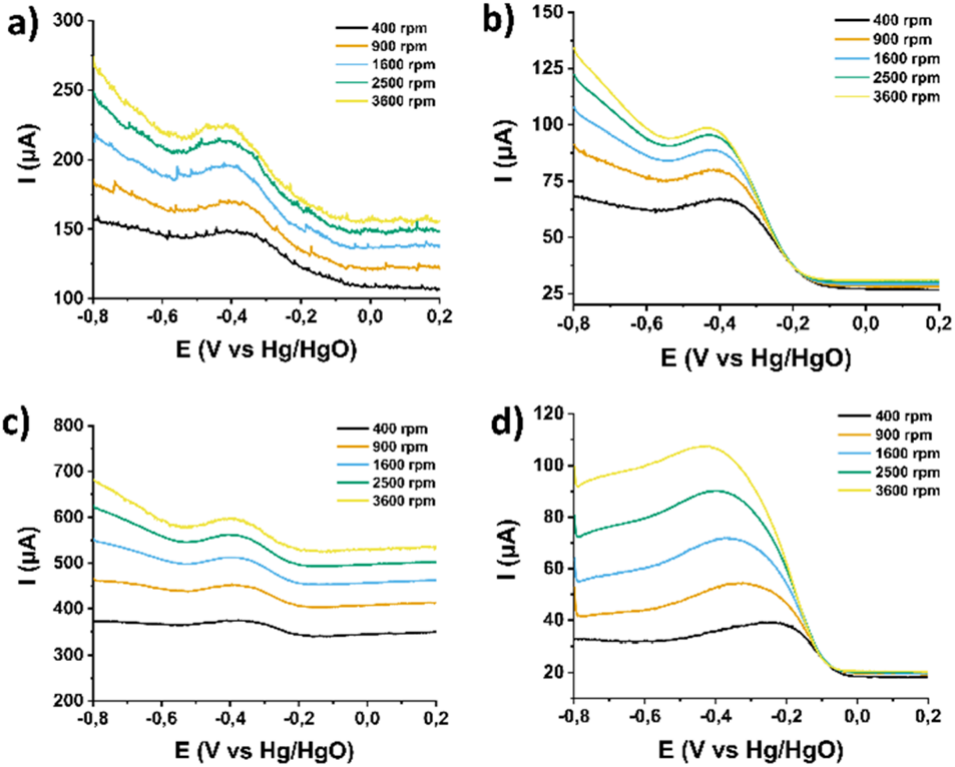
RRDE response in different rotation speeds (400–3600 rpm) for (a) (7,6)-SWCNTs, and functionalized materials (b) 1, (c) 2, and (d) 3. All assays were performed in O_2_-saturated aqueous 0.1 M KOH as electrolyte with a scan rate of 5 mV s^−1^.

The RRDE approach was applied to further investigate the kinetics of (7,6)-SWCNTs, 1, 2, and 3 (Fig. 9). Specifically, in addition to recalculating the number of transferred electrons (*n*) based on the current recorded at the ring electrode, this method provides a reliable way to quantify the amount of H_2_O_2_ produced as an intermediate or final product during the ORR.^45^ In particular, within the potential range of −0.2 to −0.5 V (*vs.* Hg/HgO), the number of transferred electrons and the percentage of H_2_O_2_ for material 3 were determined to be 3.4 and 55%, respectively. This confirms that the catalyst follows a mixed pathway, where part of the O_2_ is reduced *via* the 4e^−^ pathway to H_2_O and part *via* the 2e^−^ pathway to H_2_O_2_. Comparing these results with the values in Table S1† for the precursor materials (7,6)-SWCNTs, 1, and 2, a clear mechanistic shift is observed in material 3. Indeed, selective H_2_O_2_ production is recorded for all three precursor materials ((7,6)-SWCNTs, 1 and 2), reinforcing the findings of the K–L analysis. At this point, it is important to highlight that introducing Ru^3+^ from material 1 to material 2 caused minimal change in the catalytic activity of the terpyridine-based electrocatalyst, with a slight negative impact, as reflected in the various electrocatalytic parameters of Table S1.† In other words, the introduction of the metal does not improve the 2e^−^ ORR process, suggesting that Ru^3+^ does not participate in the electrocatalytic reaction mechanism leading to H_2_O_2_ production.

**Fig. 10:**
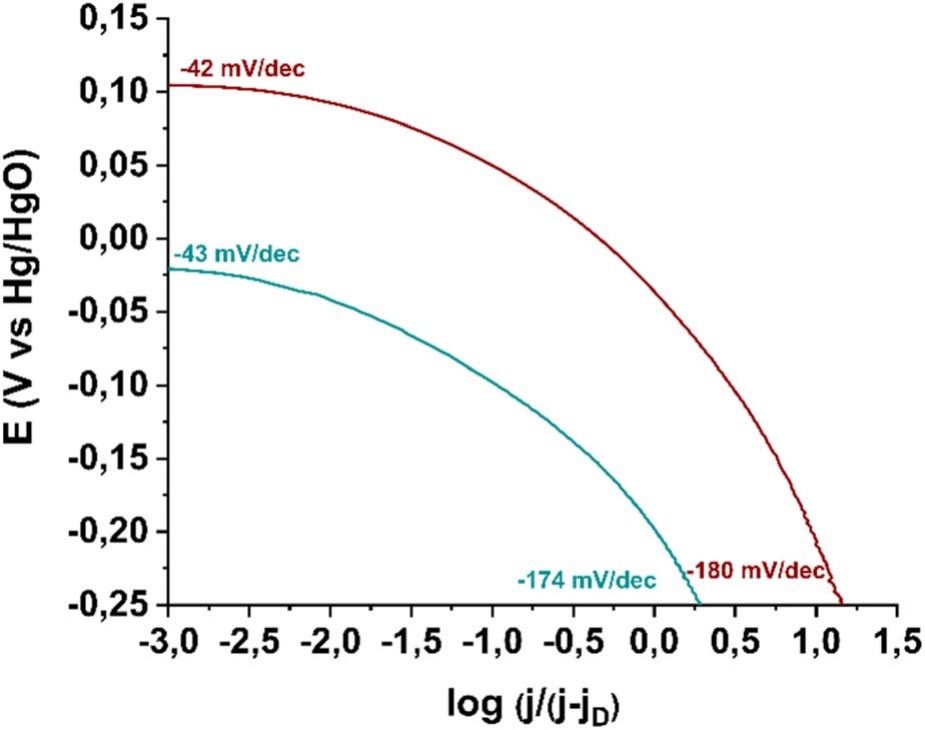
Tafel plots of electrocatalyst 3 (green) in comparison with benchmark electrocatalyst Pd/C (wine).

Next, in order to gain insights into the rate-determining step of the ORR in the 4e^−^ pathway, we constructed Tafel plots for material 3 as well as for the commercially available Pd/C catalyst. As shown in Fig. 10, in the low current density region (Region I), electrocatalyst 3 exhibits a Tafel slope of −43 mV dec^−1^, which is very close to that of the reference electrocatalyst Pd/C (−42 mV dec^−1^). A Tafel slope near −60 mV dec^−1^ in this region indicates that the reaction is governed by oxide surface coverage (physisorbed Temkin mechanism).^46^ In the high current density region (Region II), a Tafel slope of −174 mV dec^−1^ is recorded, while for the commercial Pd/C catalyst, the corresponding value is −180 mV dec^−1^. A higher Tafel slope in Region II is attributed to a mechanistic shift in the reaction, as oxide surface coverage ceases to exist at these potentials. Specifically, a Tafel slope around −120 mV dec^−1^ suggests that ORR efficiency is limited by the first electron transfer to the oxygen molecule (Langmuir physisorption mechanism), while a Tafel slope near −200 mV dec^−1^ indicates that molecular oxygen physisorption on the electrocatalyst surface determines the reaction progress.^47,48^ Thus, electrocatalyst 3 catalyzes the reaction *via* the Langmuir mechanism in Region II.

Based on the above findings, the electrocatalytic activity of the final material 3, incorporating the divalent Ru substituent, is governed by a balanced competition between two reaction mechanisms. Ultimately, electrocatalyst 3 exhibits the optimal electrocatalytic performance both kinetically and thermodynamically (Table S1†). For a comparison of the electrocatalytic parameters of material 3 with related CNT-based catalysts functionalized with molecular complexes, please see Table S2 at ESI.† Although the improvements of electrocatalyst 3 in onset potential and half-wave potential are only slight compared to the pristine (7,6)-SWCNTs before chemical modification, its ability to shift the reaction mechanism from 2e^−^ pathway toward the 4e^−^ pathway compensates for the fact that the covalent attachment deteriorates the ORR performance.

**Fig. 11:**
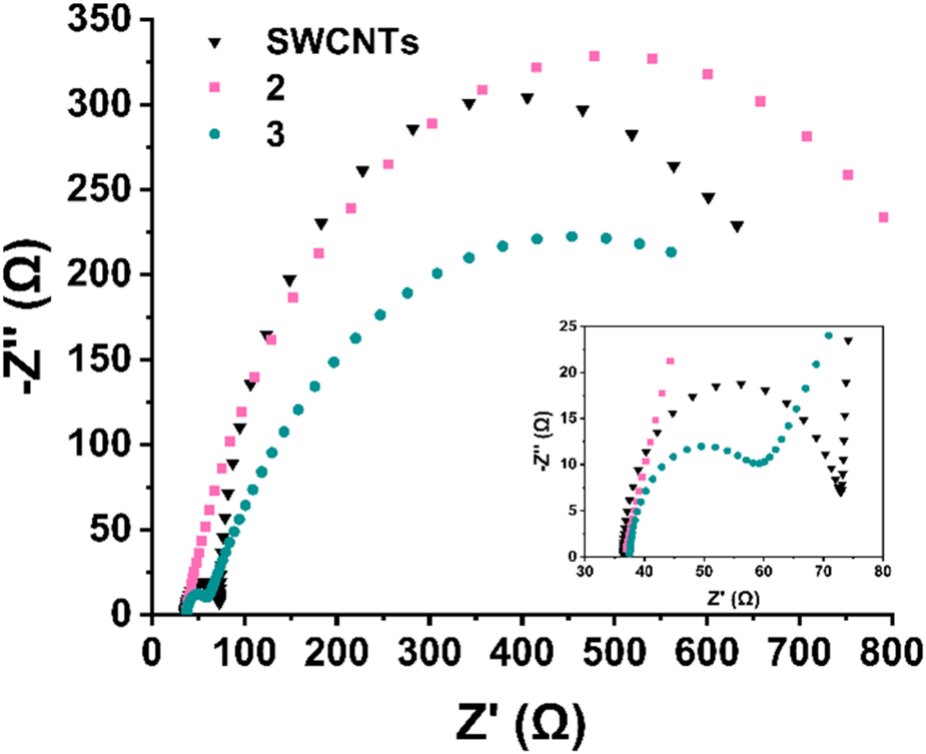
(a) Nyquist plots and corresponding equivalent circuit fits for SWCNTs, 2 (Ru^3+^), and 3 (Ru^2+^) recorded at −0.21 V *vs.* Hg/HgO in O_2_-saturated 0.1 M KOH. Material 2 displays a single semicircle, modeled using a Randles circuit with a constant phase element (CPE), indicative of a single charge-transfer process. In contrast, SWCNTs and material 3 exhibit two distinct semicircles (inset), reflecting the presence of two interfacial processes. These were fitted using an extended circuit incorporating an additional *R*_ads_‖*C*_ads_ branch to account for the adsorption of oxygen reduction intermediates. The smaller high-frequency arc corresponds to *R*_ads_ (adsorption), while the larger low-frequency arc corresponds to *R*_ct_ (charge transfer). Equivalent circuit diagrams are depicted in Fig. S2.† Inset shows the magnified *Z*′ area 30–80 Ω, where the first semicircles for SWCNTs and 3 are observed (*R*_ads_‖*C*_ads_).

In order to evaluate the entirely different electrocatalytic behaviour between Ru-functionalized materials 2 and 3, further mechanistic studies were conducted. To gain deeper insight into the interfacial and kinetic processes governing the ORR, electrochemical impedance spectroscopy (EIS) was performed at 10 mV higher overpotential than the onset potential of each material in O_2_-saturated 0.1 M KOH. It should be noted that studying and interpreting EIS analysis in ORR is a rather tricky endeavour orchestrated by difficulties in deconvoluting overlapping time constants of kinetics from O_2_ diffusion.^49^ In this study, the Nyquist plots (Fig. 11) were fitted using equivalent circuits tailored to the behaviour of each material. In Table S3,† the fitted values of all elements utilized in the fitting process are depicted for comparison purposes. Fig. S2† depicts the equivalent circuits utilized for the fitting process. In detail, electrocatalyst 2 (Ru^3+^ functionalized) exhibits a single, well-defined semicircle, consistent with a single charge-transfer process. This was modelled using a Randles circuit (Fig. S2a†) with a constant phase element (CPE), yielding a high charge transfer resistance (*R*_ct_) of 906 Ω and a CPE exponent of 0.793, reflecting the non-ideal interfacial behaviour and poor ORR activity of material 2. In stark contrast, both pristine SWCNTs and electrocatalyst 3 (Ru^2+^ functionalized) display two distinguishable semicircles, indicating the presence of two separate interfacial processes. Both these systems were modelled using an extended circuit incorporating an additional *R*_ads_‖*C*_ads_ branch to account for a fast, preceding electron-transfer step (Fig. S2b and c†), *i.e.* the adsorption of oxygen-containing intermediates (*e.g.*, *O_2_, *OOH).^50^ The relative arc sizes and fitted values indicate that the *R*_ads_ branch corresponds to the high-frequency (fast) process, while the larger, low-frequency arc reflects the slower charge-transfer step. In detail, for electrocatalyst 3, the adsorption resistance *R*_ads_ is 19.3 Ω and the corresponding capacitance *C*_ads_ is 7.36 μF, whereas the charge-transfer resistance *R*_ct_ is significantly higher at 716 Ω. Similarly, SWCNTs show a value of *R*_ads_ = 36.8 Ω and *C*_ads_ = 6.36 μF, while a larger *R*_ct_ of 563 Ω is recorded. Therefore, it is indicated that both materials undergo an initial adsorption-controlled step followed by rate-limiting electron transfer. The presence of the *R*_ads_‖*C*_ads_ feature, especially in electrocatalyst 3, supports the involvement of *OOH-type intermediates and aligns with the enhanced 4e^−^ ORR selectivity revealed by RRDE analysis. The low *R*_ct_ value and elevated Warburg *Y*_0_ (23.1 mMho·s^1/2^) for electrocatalyst 3 further confirm its superior charge-transfer and mass transport characteristics. While SWCNTs exhibit a slightly lower charge transfer resistance (*R*_ct_ = 563 Ω) compared to electrocatalyst 3 (*R*_ct_ = 716 Ω), this alone does not reflect catalytic superiority. Instead, electrocatalyst 3 demonstrates a significantly lower adsorption resistance (*R*_ads_ = 19.3 Ω *vs.* 36.8 Ω for SWCNTs), indicating more facile adsorption of oxygen reduction intermediates and particularly of *OOH species, a key step in the 4e^−^ pathway. Additionally, the adsorption capacitance (*C*_ads_ = 7.36 μF) and higher Warburg *Y*_0_ (23.1 mMho·s^1/2^) in 3 suggest increased surface coverage and enhanced mass transport characteristics. These trends are consistent with the structural characteristics of 3, where the terpyridine-Ru^2+^ complex introduces both electronic activation and favourable binding environments for ORR intermediates. Although SWCNTs facilitate reasonably fast electron transfer, the less efficient adsorption process likely limits their activity to a predominantly 2e^−^ pathway. Furthermore, the increased *R*_ct_ registered for material 2, mirrors sluggish kinetics and very low overall electrocatalytic performance, despite the presence of Ru^3+^ complex. In stark contrast, the reduction to Ru^2+^ (material 3) is accompanied by drastic change in mass transport and eventually a more selective and kinetically preferred 4e^−^ ORR mechanism is supported.

In order to further evaluate the already discussed mechanistic insights, determine active surface area differences between the studied catalysts and unveil the role of the Ru^2+^ anchoring, electrochemical surface area (ECSA) measurements were carried out. For the estimation of the ECSA, cyclic voltammograms were recorded in a non-faradaic region for ORR of SWCNTs and materials 1–3, in O_2_-saturated aqueous 0.1 M KOH electrolyte, at a rotation speed of 1600 rpm and scan rates from 50 to 500 mV s^−1^ (Figure S3†). ECSA values were obtained from *C*_dl_ by plotting the Δ*j* = (*j*_a_ – *j*_c_) at a given potential *versus* the scan rate (Figure S3† insets). Pristine SWCNTs exhibited the highest ECSA (152.5 cm^2^), consistent with their intrinsic nanostructured morphology. Following covalent functionalization, material 1 exhibits a reduced ECSA of 27.75 cm^2^, likely due to partial surface coverage and π-system disruption. Metalation with Ru^3+^ results in material 2, which displays a drastically reduced ECSA of only 3.36 cm^2^ (seven times lower that its precursor), suggesting surface passivation or aggregation effects following Ru^3+^ complexation, which perfectly aligns with its overall poor ORR activity already discussed. In contrast, electrocatalyst 3 is able to preserve a moderate ECSA of 93.5 cm^2^, significantly lower than pristine SWCNTs, yet 30 times higher than 2 (Ru^3+^ functionalized). This indicates that the Ru^2+^ functionalization enables improved catalytic performance, supporting the conclusion that its ORR enhancement stems from mechanistic activation (intermediate adsorption and electronic tuning due to the Ru^2+^ presence) rather than surface area alone.

**Fig. 12:**
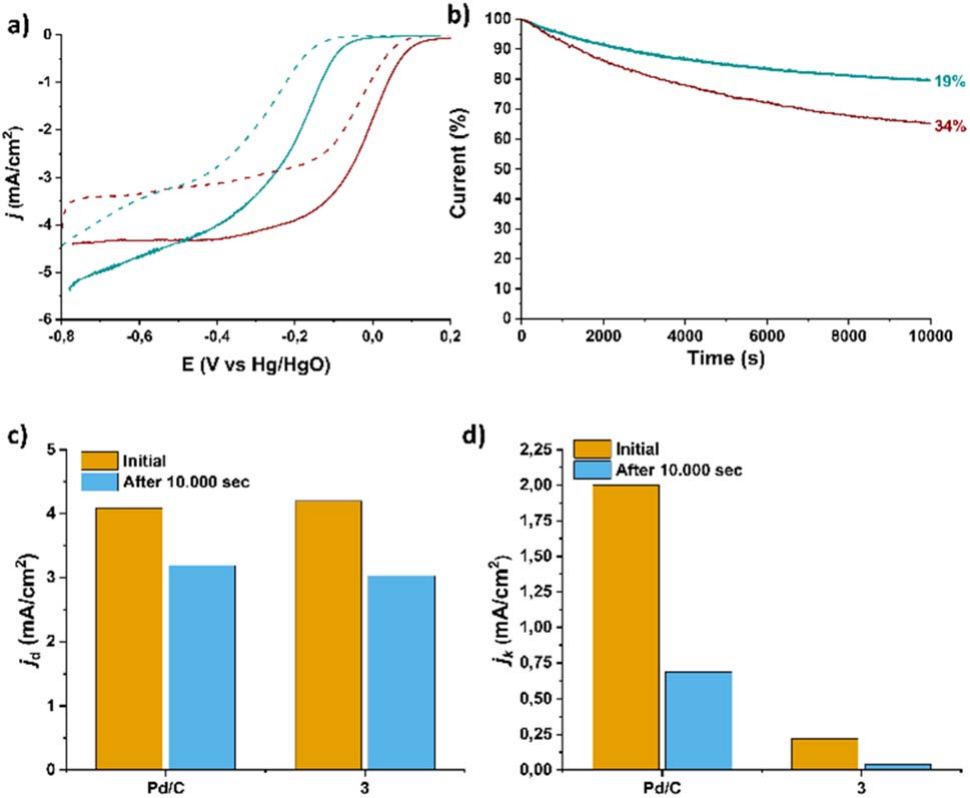
(a) LSV ORR polarization curves obtained at a rotation speed of 1600 rpm for reference electrocatalyst Pd/C (wine) and material 3 (green) before and after 10.000 s of chronoamperometric assays; (b) ORR chronoamperometric response for material 3 compared to Pd/C at −0.45 V (*vs.* Hg/HgO) for 10.000 s and (c) and (d) corresponding diffusion limited and kinetic current density values. All measurements were conducted in O_2_-saturated 0.1 M KOH electrolyte, and the corresponding electrocatalyst LSVs were recorded at a scan rate of 5 mV s^−1^. In all graphs, the current densities are normalized to the geometric electrode area.

Finally, the long-term electrochemical stability of 3, in comparison with commercially available Pd/C, was evaluated *via* chronoamperometry assays at −0.45 V *vs.* Hg/HgO for 10 000 seconds in O_2_-saturated 0.1 M KOH. As shown in Fig. 12, electrocatalyst 3 is capable of retaining 81% of its initial current, exhibiting a 19% activity loss, whereas Pd/C shows a more pronounced degradation, with a 34% decrease in current density. These results highlight the improved stability of the Ru^2+^-functionalized SWCNT electrocatalyst 3 under prolonged electrocatalytic operation. To further assess performance integrity, as well as the electrocatalyst's stability over recycling, linear sweep voltammetry (LSV) was conducted after the chronoamperometry assays. For Pd/C, a noticeable negative shift in both onset potential (from +0.064 V to +0.047 V) and half-wave potential (from −0.011 V to −0.020 V) is observed, accompanied by a significant drop in kinetic current density from 2.00 to 0.69 mA cm^−2^ after 10 000 s of operation. In contrast, 3 shows a smaller potential shift regarding both the onset (from −0.036 to −0.110 V) and half-wave potentials (from- 0.167 V to −0.252 V) after 10 000 s of recycling. In parallel, a rather moderate decrease in diffusion-limited current density (from 4.3 to 3.03 mA cm^−2^), albeit with a significant drop in kinetic current density from 0.22 to 0.04 mA cm^−2^ is observed for final electrocatalyst 3 implying possible or accumulation of reaction intermediates (*e.g.*, *OH) blocking active sites.

Overall, this study yields several highly significant conclusions regarding the fundamental design of nano-electrocatalysts based on carbon nanostructures for the ORR. Primarily, covalent chemical modification of the graphitic lattice, which disrupts sp^2^ hybridization, has detrimental effects on ORR performance in terms of onset potential. This occurs because the extensive conjugation is interrupted, hindering voltage-induced electrical conductivity, which is crucial for facilitating charge transfer at the material's surface. Consequently, the degree of chemical modification also influences electrocatalytic activity both due to the disruption of the lattice, as mentioned above, and due to the nature of the substituent immobilized on the lattice, which is then responsible to compensate for the adverse structural changes to maintain significant catalytic activity. Furthermore, this comparative study reveals a crucial insight regarding the role of precious metals in electrocatalysis. The success of an electrocatalytic reaction is often attributed solely to the presence of precious metals, regardless of the surrounding chemical environment within an electrocatalytic system. However, we conclude that the first coordination sphere of the metal substituent significantly affects ORR performance, both thermodynamically and kinetically. In other words, the mere presence of a metal center does not necessarily guarantee effective catalytic activity. Interestingly, although macroscopically the structural modification of substituents from material 2 to material 3 appears to be minor -whereas the modification from material 1 to material 2 seems substantial- the electrocatalytic results show the exact opposite trend. Introducing the metal center in material 2 (transition from 1 to 2) does not improve the catalytic performance at all. However, the addition of an extra tridentate ligand in material 3 (transition from 2 to 3) drastically enhances electrocatalytic activity and simultaneously shifts the reaction mechanism toward a more favorable and environmentally friendly (“green”) 4e^−^ pathway. Indeed, the dramatic improvement in electrocatalytic behavior from material 2 to material 3 is attributed to the change in the metal's oxidation state, which is reduced from Ru^3+^ to Ru^2+^. Specifically, it is demonstrated that Ru^3+^ does not participate in the 2e^−^ ORR mechanism for H_2_O_2_ production, whereas, in contrast, Ru^2+^ significantly promotes the 4e^−^ pathway.

## Conclusions

In conclusion, the stepwise engineering of (7,6)-SWCNTs was realized by the introduction of a terpyridine ligand on the carbon nanotube lattice, that was then metalated with Ru^3+^, followed by reduction to Ru^2+^*via* complexation with an extra terpyridine substituent. The step-by-step functionalization was monitored by UV-Vis and Raman spectroscopy as well as by TGA analysis to unveil the terpyridine loading achieved. In parallel, STEM imaging provided insights on the structural integrity of SWCNTs as well as the presence of the Ru atoms and terpyridine complex, which was also corroborated by XPS and EDS measurements. Detailed electrocatalytic investigation toward ORR reveals significant insights into the design principles of engineered nano-electrocatalysts based on carbon nanostructures. The findings highlight the critical effect of the extent of chemical modification on the electrocatalytic activity not only due to lattice disruption but also due to the nature of the substituent immobilized on the surface. Detailed RRDE assays accompanied by EIS and ECSA analysis, along with chronoamperometry study, reveal mechanistic details crucial to the understanding of such carbon-based electrocatalytic systems. The results indicate that Ru^3+^ does not actively participate in the 2e^−^ ORR pathway for H_2_O_2_ production. In contrast, Ru^2+^ significantly promotes the 4e^−^ pathway, facilitating direct water formation. This mechanistic insight highlights the importance of precise molecular engineering when designing carbon-based electrocatalysts, as subtle alterations in metal coordination can profoundly influence both catalytic efficiency and reaction selectivity. Overall, this work provides valuable guidance for the rational design of next-generation carbon-based ORR electrocatalysts, whether targeting the 2e^−^ or 4e^−^ reaction mechanism. By demonstrating how covalent functionalization, the nature of substituents, and metal coordination influence ORR performance, this study contributes to the development of more effective, high-selectivity catalysts for energy conversion applications. Future research should further explore the interplay between metal oxidation states, ligand environments, and electronic properties to optimize electrocatalytic systems for clean energy technologies.

## Author contributions

Ioanna K. Sideri: conceptualization, data curation, formal analysis, investigation, methodology, validation, visualization, writing – original draft, writing – review & editing; Raul Arenal: data curation, formal analysis, investigation, methodology, validation, funding acquisition, resources, writing – original draft; Nikos Tagmatarchis: conceptualization, funding acquisition, project administration, resources, supervision, writing – review & editing.

## Conflicts of interest

There are no conflicts to declare.

## Supplementary Material

NA-007-D5NA00281H-s001

## Data Availability

The data supporting this article have been included as part of the main article and the ESI.†
