# Supplementary material for: Terpyridine-functionalized single-walled carbon nanotubes towards selectivity in the oxygen reduction reaction
Source: Nanoscale Adv. 2025 Jun 4;7(14):4469–79. doi: 10.1039/d5na00281h (PMC12153378; doi:10.1039/d5na00281h)
Supplement: NA-007-D5NA00281H-s001 [file NA-007-D5NA00281H-s001.pdf]

Supplementary Information (SI) for Nanoscale Advances.
This journal is © The Royal Society of Chemistry 2025

ELECTRONIC SUPPLEMENTARY INFORMATION

Terpyridine-Functionalized Single-Walled Carbon Nanotubes Towards Selectivity in Oxygen Reduction Reaction

Ioanna K. Sideri,[a*] Raul Arenal,[b,c,d] and Nikos Tagmatarchis[a*]

[a] *Theoretical and Physical Chemistry Institute, National Hellenic Research Foundation, 48 Vassileos Constantinou Avenue, 11635 Athens, Greece.*

[b] *Instituto de Nanociencia y Materiales de Aragon (INMA), CSIC-U. de Zaragoza, 50009 Zaragoza, Spain*

[c] *Laboratorio de Microscopias Avanzadas (LMA), Universidad de Zaragoza, 50018 Zaragoza, Spain*

[d] *ARAID Foundation, 50018 Zaragoza, Spain*

1. Instrumentation

Mid-infrared spectra in the region 500-4500 cm^{-1} were acquired on a Fourier transform IR spectrometer (Equinox 55 from Bruker Optics) equipped with a single reflection diamond ATR accessory (DuraSamp1IR II by SensIR Technologies). Typically, 100 scans were acquired at 2 cm^{-1} resolution.

Micro-Raman scattering measurements were performed at room temperature in the backscattering geometry using a RENISHAW in Via Raman spectrometer equipped with a CCD camera and a Leica microscope. Measurements were taken with 10 seconds of exposure times and laser power of ~0.3 mW cm^{-2} to prevent overheating and damage of the basal plane. The laser spot was focused on the sample surface using a long working distance 50× (L50) objective. Raman spectra were collected on numerous spots on the sample and recorded with a Peltier cooled CCD camera. The data were collected and analyzed with Renishaw Wire and Origin software. The intensity ratio I_D/I_G was obtained by taking the peak intensities following any baseline corrections. For the mapping recordings, 5-10 areas of 121 acquisition points each were scanned for every sample and we present here a representative one close to the total average with respect to the intensity ratio I_D/I_G. The data were collected and analyzed with Renishaw Wire and Origin software.

Thermogravimetric analysis was performed using a TGA Q500 V20.2 Build 27 instrument by TA in an inert atmosphere of nitrogen (purity >99.999%). In a typical experiment, 2 mg of the material was placed in the platinum pan and the temperature was equilibrated at 40°C. Subsequently, the temperature was increased to 900°C with a rate of 10°C/min and the mass changes were recorded as a function of temperature.

UV-Vis absorption spectra were recorded on a PerkinElmer (Lambda 19) UV-Vis-NIR spectrophotometer.

Scanning transmission electron microscopy (STEM) studies were developed using a probe-aberration-corrected Thermo Fisher Scientific Titan Low Base microscope. This STEM instrument is equipped with a high-brightness gun (X-FEG). All measurements were performed at an acceleration voltage of 120 kV.

X-ray photoelectron spectroscopy (XPS) measurements were performed in a Kratos Axis SUPRA spectrometer employing a monochromatic Al Kα (1486.6 eV) X-ray source. Survey spectra were obtained with an energy step of 1 eV and 160 eV analyzer pass energy. The spectra were analyzed using CASA XPS software. Shirley baseline was used to subtract the

background for quantification purposes. The spectra were calibrated using the B.E. of the C 1s peak at 284.5 eV due to the use of the charge neutralizer during the spectra acquisition.

High-Resolution transmission electron microscopy (HRTEM) imaging and energy dispersive X-*ray spectroscopy (EDS)* analyses have been performed in a doble-aberration-corrected Thermo Fisher Scientific Titan Cube working at 300 kV. EDS measurements were carried out using the Super-X system consisting in 4 window-less SDD detectors, which provide a solid angle ≈ 0.7 srad.

Oxygen Reduction Reaction (ORR) studies were carried out on an Autolab PGSTAT128 N potentiostat/galvanostat equipped with a dual mode bipotentiostat (BA module). The catalyst ink was prepared by dispersing 4.0 mg of the catalytic powder in 1 mL mixture of deionized water, isopropanol, and 5% Nafion (v/v/v = 4:1:0.02) and sonicated for 30 minutes prior use. Before casting the electrocatalytic ink on the electrode's surface, the working electrode was polished with 6, 3, and 1 μm diamond pastes, rinsed with deionized water, and sonicated in double-distilled water. Afterward, 8.5 μL aliquots of the electrocatalyst were casted on the electrode surface and were left to dry at room temperature. The working electrode (Autolab RRDE electrode tip) consisted of a Teflon-embedded glassy carbon (GC) disk/Pt ring rotating assembly (5 mm electrode disk diameter, 375 μm electrode gap with collection efficiency N = 0.249). A platinum wire was used as the counter electrode, while an Hg/HgO (0.1 M KOH) was used as the reference electrode. The ORR measurements took place at room temperature in O_2-saturated aqueous 0.1 M KOH electrolyte. Linear sweep voltammetry (LSV) measurements on the disk electrode were conducted from −0.8 to +0.2 V vs. Hg/HgO at different rotation rates with a scan rate of 5 mV s^{-1}. At the ring, the potential was fixed at +1.0 V vs. Hg/HgO and the current response was recorded. The kinetic current densities (j_k) were calculated using the Koutecký–Levich (K–L) equation:

$$\frac{1}{j} = \frac{1}{j_d} + \frac{1}{j_k}$$

where j_d and j_k are the experimentally measured and the diffusion-limited current density, respectively. The number of electrons transferred in the reduction of one O_2 molecule (n) can be determined by modifying the K–L equation as follows:

$$\frac{1}{j} = \frac{1}{j_d} + \frac{1}{j_k} = \frac{1}{B\omega^{\frac{1}{2}}} + \frac{1}{j_k}$$

where ω is the angular velocity and B is the K–L slope given by the following equation:

$$B = 0.20nF C_0 D_0^{\frac{2}{3}} v^{\frac{1}{6}}$$

Here, n is the electron transfer number, F is the Faraday constant (F = 96 485 C mol^{-1}), D_0 is the diffusion coefficient of O_2 (D_0 = 1.9 × 10^{-5} cm^2 s^{-1}), ν is the kinematic viscosity of the solution (ν = 0.01 cm2 s^{-1}), and C_0 is the concentration of dissolved O_2 in the solution (C_0 = 1.2 × 10^{-6} mol cm^{-3}). The constant of 0.2 is adopted when the rotation speed is expressed in revolutions per minute (rpm). Tafel plots (potential vs. log[$j/(j_d - j)$]) were calculated in the mixed kinetic–diffusion region at a single electrode rotation rate (ω = 1600 rpm). Based on the RRDE data, the n value and the percentage (%) of the produced H_2O_2 can be determined using the following equations:

$$n_{\mathrm{RRDE}} = 4 * \frac{I_d}{I_d + \frac{I_r}{N_c}}$$

$$(p)\ (\%) = 2 * \frac{\frac{I_r}{N_c}}{I_d + \frac{I_r}{N_c}}$$

where I_d is the current of the disk electrode, Iring is the current of the ring electrode, and N_c is the collection efficiency of the Pt ring, which was provided as 0.249 by the manufacturer. The capacitance values were calculated from the CV curves obtained in N_2-saturated aqueous 0.1 M KOH according to the following equation:

$$C = \int \frac{IdV}{\Delta V} \times v$$

where C (F) is the specific capacitance; $\int IdV(C)$ is the integrated area of the CV curve; ΔV (V) is the potential window and v is the scan rate (V s^{-1}).

Chronoamperometric measurements were probed at -0.45 V versus Hg/HgO for 10000 sec. Electrochemical impedance spectroscopy (EIS) measurements were conducted in the frequency range between 10^{-1} and 10^5 Hz with an AC amplitude of 0.01 V at applied DC potentials being at the low overpotential region (typically, EIS measurements were performed

at 10 mV higher overpotential than the onset potential of each material). Analysis of the impedance data was carried out by the NOVA 2.0 software. For the estimation of the ECSA, cyclic voltammograms were recorded in a non-Faradaic region at scan rates of 50, 100, 200, 300, 400, and 500 mV/s for all tested materials. ECSA $= C_{dl}/C_s$, where C_{dl} stands for the electrochemical double-layer capacitance, while C_s is the specific capacitance of a flat surface with 1 cm^2 of real surface area with the value assumed to be 40 $\mu F/cm^2$ for the flat electrode. ECSA values were obtained from C_{dl} by plotting the $\Delta j = (j_a - j_c)$ at a given potential versus the scan rate, as stated in the equation $C_{dl} = d(\Delta j)/2\ dV_b$.

2. Experimental

- **Synthesis of terpyridine ligands 4,[1,2] 5[3] and 6.[4]**

The terpyridine ligands **4**, **5** and **6** were synthesized according to literature procedures based on the synthetic scheme below.[1-4]

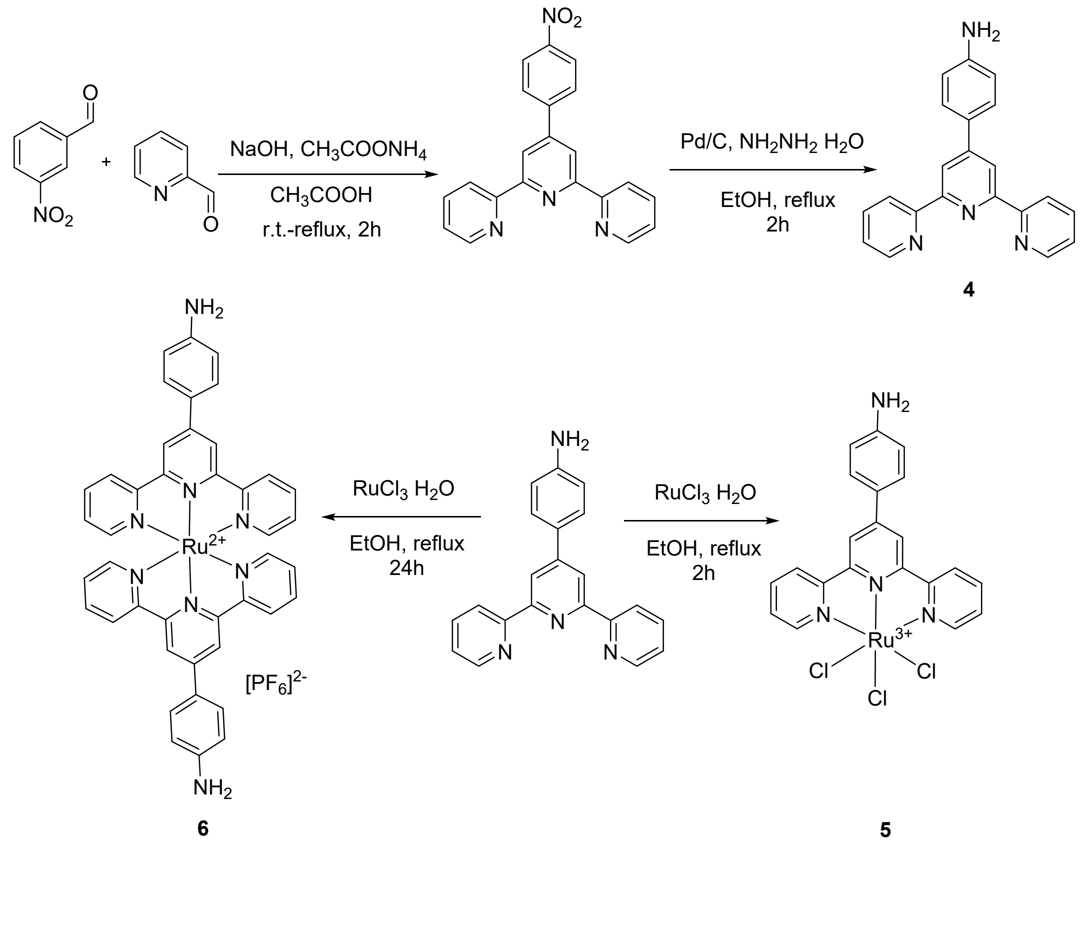

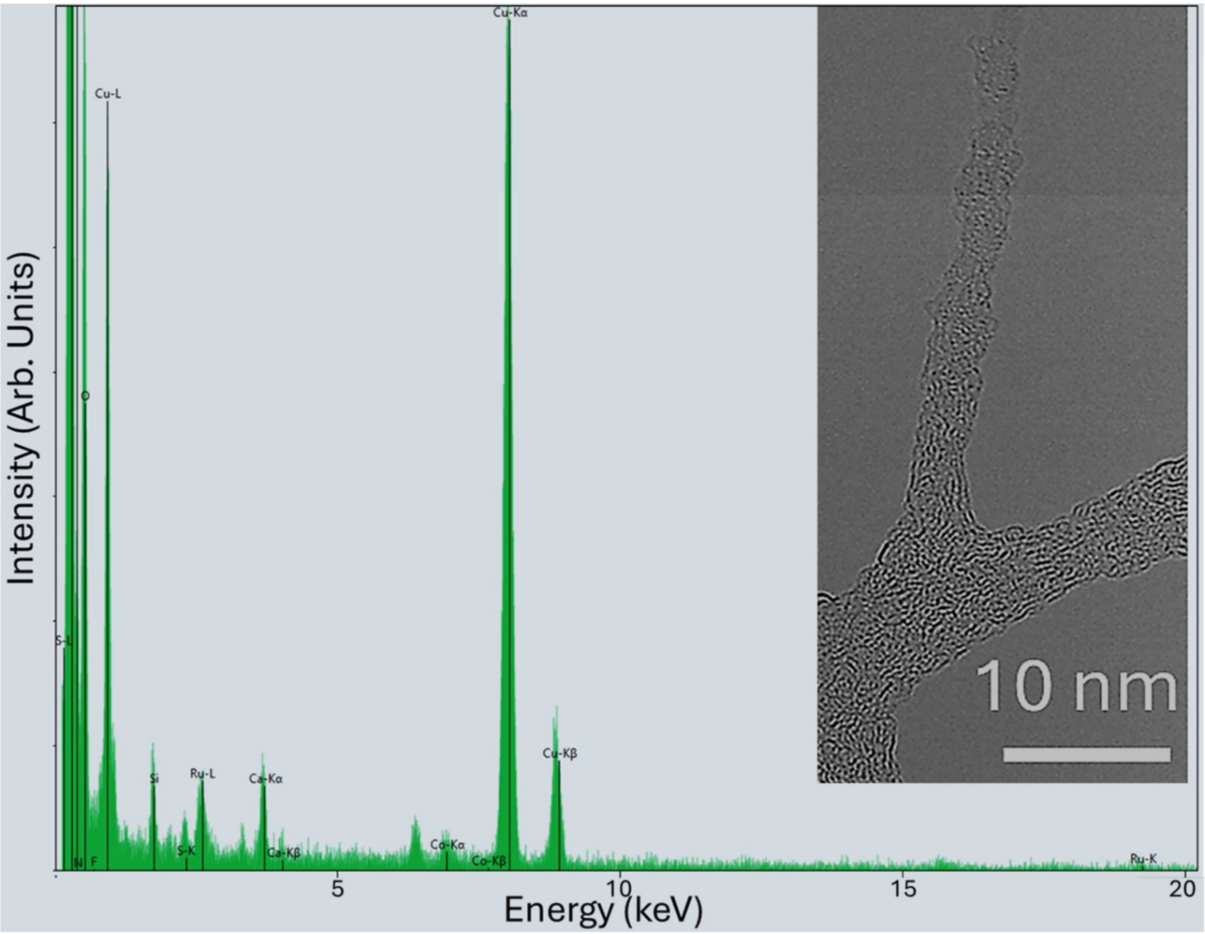

Figure S1. EDS spectrum of material **3** recorded in the region despicted in the HR-TEM image (in insert), where SWCNTs enrobed with the Ru-moities are visible. Ru signal is clearly visible in this spectrum, confirming the presence of these structures.

Table S1. ORR values of electrocatalytic parameters for materials **1**, **2** και **3** in comparison with benchmark catalyst Pd/C and precursor SWCNTs.

	η **(V)**	$\mathbf{E_{1/2}}$ **(V)**	j_d **(mA/cm²)**[a]	j_k **(mA/cm²)**[b]	**Tafel slopes (mV/dec)**	n_{KL}	n_{RRDE}	**%H_2O_2**	**ECSA (cm²)**
**Pd/C**	+0.064	-0.011	4.09	2	-42/ -180	3.6-3.7	4	32	-
**Pd/C***	+0.047	-0.02	3.19	0.69	-	-	-	-	-
**SWCNTs**	-0.056	-0.183	2.4	0.29	-	2.0-2.1	1.7	96	152.5
**1**	-0.144	-0.280	1.5	0.02	-	1.5-1.7	1.8	100	27.75
**2**	-0.164	-0.276	1.3	0.12	-	2.0-2.3	1.5	97	3.36
**3**	-0.036	-0.167	4.3	0.22	-43/ -174	3.5-3.7	3.4	55	93.5
**3***	-0.11	-0.252	3.03	0.04	-	-	-	-	-

[a]At 1600 rpm rotation rate (at ~-0.45 V vs. Hg/HgO). [b]Calculated at -80 mV vs. Hg/HgO using the K–L equation. *After 10000 s of the chronoamperometric experiment.

Table S2. Summary of electrocatalytic parameters for the oxygen reduction reaction (ORR) of material **3** (this work) compared to selected CNT-based molecular electrocatalysts reported in the literature. Data include onset (η) and half-wave potentials (**$E_{1/2}$** vs RHE), number of electrons transferred (n), as measured from RRDE experiments. The table is intended for indicative comparison only, as the focus of the present work lies in mechanistic and kinetic analysis rather than performance benchmarking.

**Electrocatalyst**	**η (V vs RHE)**	**$E_{1/2}$ (V)**	**n (RRDE)**	**Reference**
**Material 3**	0.89	0.76	3.4	This work
**FeCoN-MWCNT**	0.93	0.84	3.95	5
**CoPc/MWCNT**	0.91	0.80	3.9	6
**Metallo-corrole/CNT**	0.92	0.82	3.9	7
**MWCNT-LPd**	0.94	0.83	3.8	8
**CoNi@NCNT/NF**	0.97	0.85	4	9

All potentials are converted to RHE scale when not originally provided as such. Electrolyte in all studies is 0.1 M KOH.

Table S3. ORR electrochemical impedance spectroscopy (EIS) parameters for SWCNTs and Ru-functionalized materials **2** and **3.** The EIS measurements were performed in O_2-saturated 0.1 M KOH electrolyte at ~at 10 mV higher overpotential than the onset potential of each material. In Figure S2 the respective equivalent circuit diagrams utilized for the fitting, are depicted.

**Element**	**SWCNTs**	**2**	**3**
R_s	36.4 Ω	36.6 Ω	37.4 Ω
R_{ads}	36.8 Ω	-	19.3
C_{ads}	6.36 μF	-	7.36 μF
R_{ct}	563 Ω	906 Ω	716 Ω
C_{dl}/CPE Y_0	693 μF	29.6 μMho $s^{1/2}$	1.01 mMho s^N, N = 0.674
**Warburg** Y_0	11.1 mMho $s^{1/2}$	11.4 mMho $s^{1/2}$	23.1 mMho $s^{1/2}$

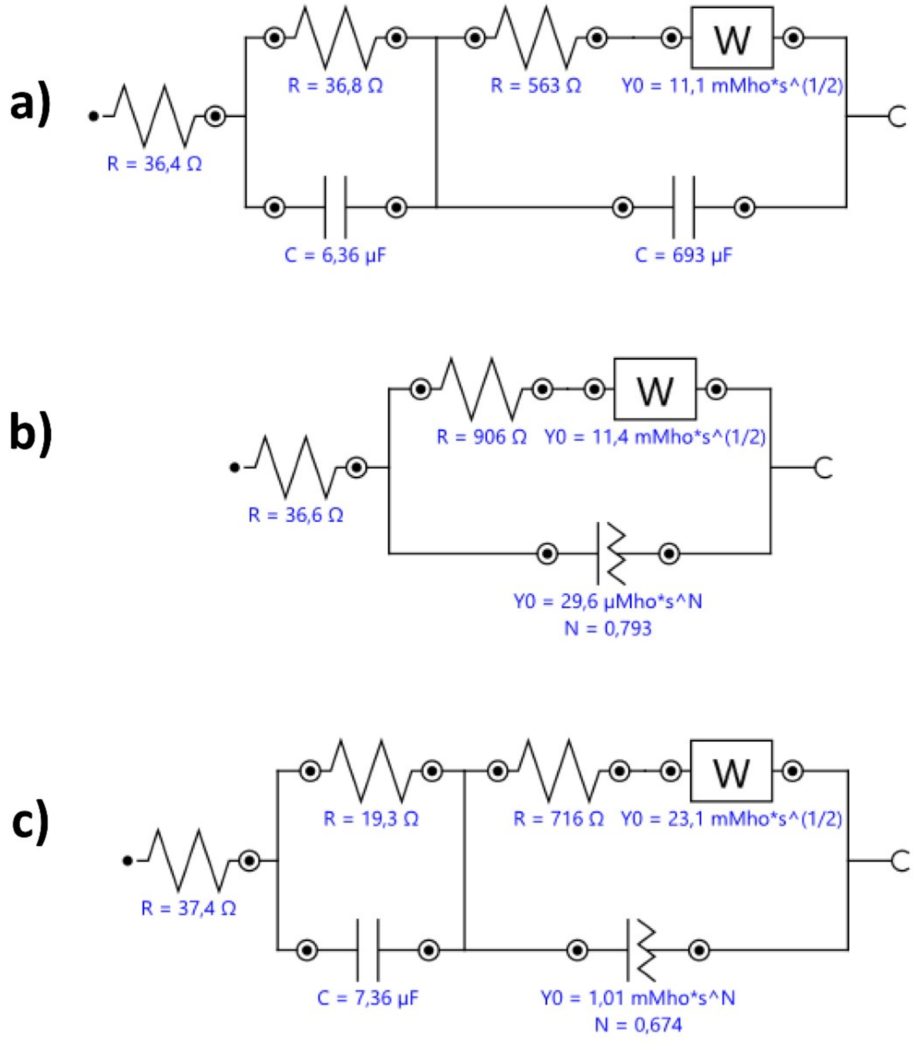

Figure S2. Equivalent circuit diagrams and respective fitted values for a) SWCNTs (Randles circuit with an extra Rads || Cads branch to account for the adsorption of oxygen reduction intermediates), b) **2** (Ru^{3+}-functionalized) (Randles circuit with a constant phase element (CPE)), and c) **3** (Ru^{2+}-functionalized) (modified Randles circuit with a constant phase element (CPE) and an extra Rads || Cads branch to account for the adsorption of oxygen reduction intermediates).

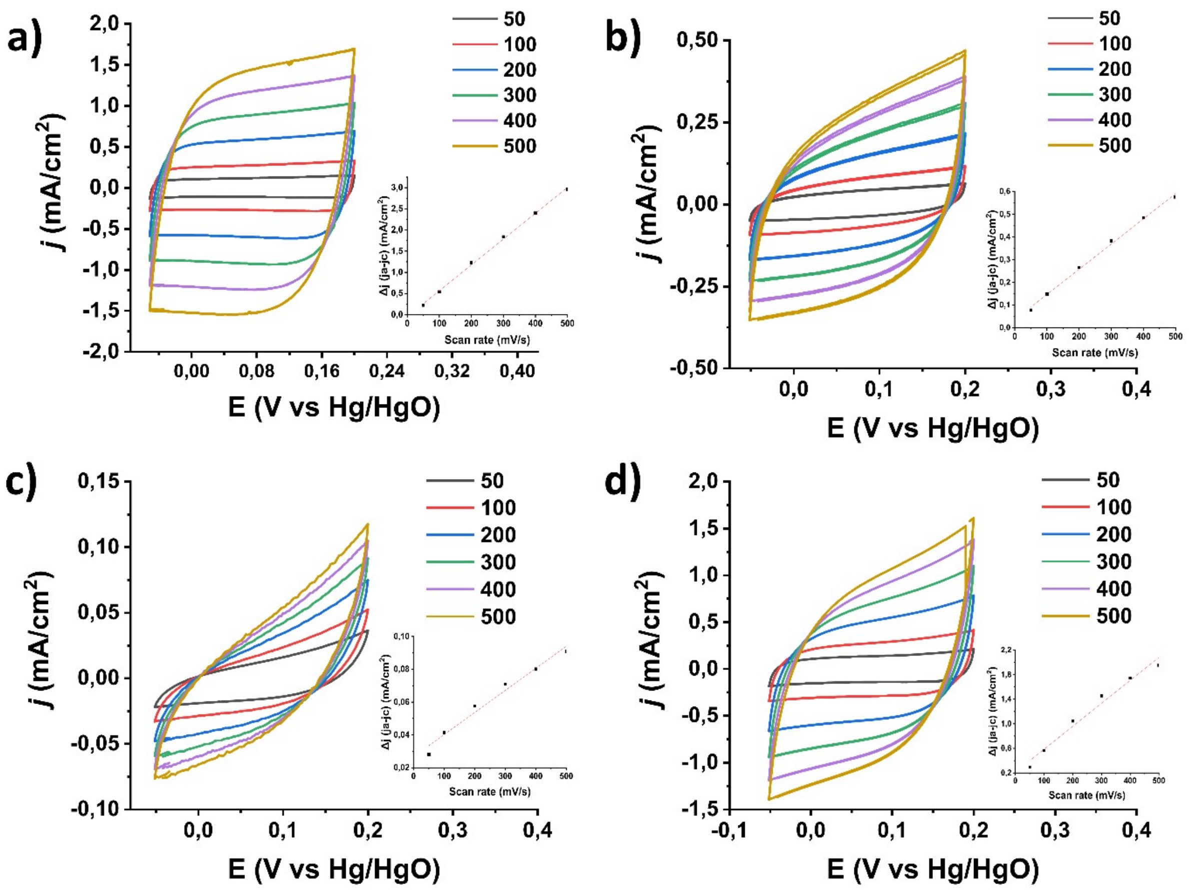

Figure S3. Cyclic voltamographs for ORR of a) SWCNTS, b) **1**, c) **2**, d) **3**, in a O_2-saturated aqueous 0.1 M KOH electrolyte, at a rotation speed of 1600 rpm and scan rates from 50 to 500 mV/s obtained in a non-Faradaic region. Inset: Scan rate dependence of the current densities for the corresponding materials.

References

1. Lainé, P.; Bedioui, F.; Ochsenbein, P.; Marvaud, V.; Bonin, M.; Amouyal, E. A New Class of Functionalized Terpyridyl Ligands as Building Blocks for Photosensitized Supramolecular Architectures. Synthesis, Structural, and Electronic Characterizations. *J. Am. Chem. Soc.* **2002**, *124*, 1364–1377.
2. Cave, G. W. V.; Raston, C. L. Efficient Synthesis of Pyridines via a Sequential Solventless Aldol Condensation and Michael Addition. *J. Chem. Soc. Perkin 1* **2001**, No. 24, 3258–3264.
3. Frath, D.; Nguyen, V. Q.; Lafolet, F.; Martin, P.; Lacroix, J. C. Electrografted Monolayer Based on a Naphthalene Diimide-Ruthenium Terpyridine Complex Dyad: Efficient Creation of Large-Area Molecular Junctions with High Current Densities. *Chem. Commun.* **2017**, *53*, 10997–11000.
4. Salpage, S. R.; Lanzetta, R. C.; Zhou, Y.; Wang, J. C.; Albrecht-Schmitt, T. E.; Hanson, K. Wavelength Selective Separation of Metal Ions Using Electroactive Ligands. *Chem. Commun.* **2018**, *54*, 7507–7510.
5. Kumar, Y.; Kibena-Põldsepp, E.; Kozlova, J.; Rähn, M.; Treshchalov, A.; Kikas, A.; Kisand, V.; Aruväli, J.; Tamm, A.; Douglin, J. C.; Folkman, S. J.; Gelmetti, I.; Garcés-Pineda, F. A.; Galán-Mascarós, J. R.; Dekel, D. R.; Tammeveski, K. Bifunctional Oxygen Electrocatalysis on Mixed Metal Phthalocyanine-Modified Carbon Nanotubes Prepared via Pyrolysis, *ACS Appl. Mater. Interfaces* **2021**, *13*, 41507-41516.
6. Xu, Z.; Hejun, L.; Cao, G.; Zhang, Q.; Li, K.; Zhao X. Electrochemical performance of carbon nanotube-supported cobalt phthalocyanine and its nitrogen-rich derivatives for oxygen reduction, *J. Mol. Catal. A Chem.* **2011**, *335*, 89-96.
7. Raggio, M.; Mecheri, B.; Nardis, S.; D'Epifanio, A.; Licoccia, S.; Paolesse, R. Metallo-Corroles Supported on Carbon Nanostructures as Oxygen Reduction Electrocatalysts in Neutral Media, *Eur. J. Inorg. Chem.* **2019**, *44*, 4760-4765.
8. Monini, V.; Bonechi, M.; Bazzicalupi, C.; Bianchi, A.; Gentilesca, P.; Giurlani, W.; Innocenti, M.; Meoli, A.; Romano, G. M.; Savastano, M. Oxygen reduction reaction (ORR) in alkaline solution catalysed by an atomically precise catalyst based on a Pd(ii) complex supported on multi-walled carbon nanotubes (MWCNTs). Electrochemical and structural considerations, *Dalton Trans*. **2024**, *53*, 2487-2500.

9. Niu, W.; Pakhira, S.; Marcus, K.; Li, Z.; L. Mendoza-Cortes, J.; Yang Y. Apically Dominant Mechanism for Improving Catalytic Activities of N-Doped Carbon Nanotube Arrays in Rechargeable Zinc–Air Battery, *Adv. Energy Mat*er. **2018**, *8*, 1800480.
